# Microglial SIRT1 activation attenuates synapse loss in retinal inner plexiform layer via mTORC1 inhibition

**DOI:** 10.1186/s12974-023-02886-8

**Published:** 2023-09-05

**Authors:** Ke Yao, Qianxue Mou, Xiaotong Lou, Meng Ye, Bowen Zhao, Yuanyuan Hu, Jing Luo, Hong Zhang, Xing Li, Yin Zhao

**Affiliations:** 1 Department of Ophthalmology, Tongji Hospital, Tongji Medical College, Huazhong University of Science and Technology, Wuhan, 430030 China; 2 Institute of Reproductive Health, Center for Reproductive Medicine, Tongji Medical College, Huazhong University of Science and Technology, Wuhan, China; 3 Department of Anesthesiology, Tongji Hospital, Tongji Medical College, Huazhong University of Science and Technology, Wuhan, 430030 China

**Keywords:** Microglia, Synaptic loss, SIRT1, mTORC1, Deacetylation

## Abstract

**Background:**

Optic nerve injury (ONI) is a key cause of irreversible blindness and triggers retinal ganglion cells (RGCs) change and synapse loss. Microglia is the resistant immune cell in brain and retina and has been demonstrated to be highly related with neuron and synapse injury. However, the function of Sirtuin 1 (SIRT1), a neuroprotective molecule, in mediating microglial activation, retinal synapse loss and subsequent retinal ganglion cells death in optic nerve injury model as well as the regulatory mechanism remain unclear.

**Method:**

To this end, optic nerve crush (ONC) model was conducted to mimic optic nerve injury. Resveratrol and EX527, highly specific activator and inhibitor of SIRT1, respectively, were used to explore the function of SIRT1 in vivo and vitro. Cx3Cr1-Cre^ERT2^/Raptor^F/F^ mice were used to delete Raptor for inhibiting mammalian target of rapamycin complex 1 (mTORC1) activity in microglia. HEK293 and BV2 cells were transfected with plasmids to explore the regulatory mechanism of SIRT1.

**Results:**

We discovered that microglial activation and synapse loss in retinal inner plexiform layer (IPL) occurred after optic nerve crush, with later-development retinal ganglion cells death. SIRT1 activation induced by resveratrol inhibited microglial activation and attenuated synapse loss and retinal ganglion cells injury. After injury, microglial phagocytosed synapse and SIRT1 inhibited this process to protect synapse and retinal ganglion cells. Moreover, SIRT1 exhibited neuron protective effects via activating tuberous sclerosis complex 2 (TSC2) through deacetylation, and enhancing the inhibition effect of tuberous sclerosis complex 2 on mammalian target of rapamycin complex 1 activity.

**Conclusion:**

Our research provides novel insights into microglial SIRT1 in optic nerve injury and suggests a potential strategy for neuroprotective treatment of optic nerve injury disease.

**Supplementary Information:**

The online version contains supplementary material available at 10.1186/s12974-023-02886-8.

## Introduction

Visual information is sent from the retina to central visual targets through the optic nerve formed of retinal ganglion cells’ (RGCs) axons [1]. The dendrites of RGCs have a profound impact on integrating neuronal input from photoreceptors by intermediate neuronal cells including bipolar and amacrine, via synapses. Importantly, it is increasingly recognized that injury to RGC axons triggers rapid changes in dendrites, including dendritic shrinkage, reduced complexity, and synapse loss [2–6]. A better understanding of how to attenuate RGC dendrites or synapse loss in early neurodegenerative conditions, such as in glaucoma, might provide new insights into disease progression, while informing the development of novel therapies to prevent vision loss [4].

Components of the innate immune system were reported to regulate synapse formation and stabilization in central nervous system [7, 8]. Microglia, the predominant immune cells within the brain parenchyma and retina, have been increasingly implicated in synapse remodeling [8–12]. It was reported that pharmacological depletion of microglia causes post-injury accumulation of synaptic debris, suggesting that microglia are the dominant post-injury phagocytes [10]. However, it is still unclear whether and how microglia participate in RGC dendrites or synapse loss during neurodegenerative conditions, such as optic nerve injury (ONI).

Sirtuin 1 (SIRT1), a nicotinamide adenine dinucleotide (NAD+)-dependent deacetylase, deacetylates histones and non-histone proteins [13, 14]. Researchers found SIRT1 is reduced with the aging of microglia and that microglial SIRT1 deficiency has a causative role in aging- or tau-mediated memory deficits via epigenetic regulation of IL-1β in mice [15]. Resveratrol is a natural activator of SIRT1 [14]. Several neural cell experiments have demonstrated that resveratrol suppresses inflammatory cytokines via inhibition of NF-κB transcriptional activity [16, 17]. Mammalian target of rapamycin complex 1 (mTORC1) is a molecule complex, consisting of mTOR, mammalian lethal with SEC13 protein 8 (mLST8), regulatory-associated protein of mTOR (raptor), DEP domain-containing protein 6 (DEPTOR) and GTPase-activating protein for Rheb (Rheb-GTP), etc. [18]. Many researches pointed that mTORC1 pathway disruption ameliorates inflammation following brain or retina injury via a shift in microglia phenotype from M1 type to M2 type or downregulation of inflammatory factors, beneficial for neurodegenerative process [19–21]. Tuberous sclerosis complex 1 (TSC1) and TSC2 was considered as negatively control mTORC1 activity by forming a heterodimer that acts as a GTPase-activating protein for Rheb [22, 23]. Previous study has reported mTORC1 as a target of SIRT1, since SIRT1 could inhibit ribosomal S6 kinase (S6K1) acetylation, and therefore activates mTORC1 signaling [24]. Besides, cartilage ablation of SIRT1 was found to cause inhibition of growth plate chondrogenesis by hyperactivation of mTORC1 signaling [25]. Recently, our team discovered that microglial SIRT1-mTORC1 pathway could modulate the degeneration of RGC axons after ONC in mice [26]. Previous study reported that C1q protein response was consistently the strongest in nerve injury site post-ONC and a relatively weak response in the retina [27]. And the crush injury site is far from retinal RGC and synapse. Hence, the potential function of SIRT1–mTORC1 pathway in retinal microglia to regulate RGC and synapse loss needs to be clarified. Furthermore, the molecular mechanism by which SIRT1 controls mTORC1 signaling in microglia has not yet been fully elucidated.

In this study, we aim to elucidate the involvement of SIRT1–mTORC1 pathway in microglia, especially in ONI-induced RGC and synapse loss by using resveratrol as well as microglial Raptor knockout mice. Experiments in vitro were conducted to explore a novel mechanism by which SIRT1 modulates mTORC1 pathway via post-transcriptional modification of TSC2. Our findings suggested a novel neuroprotective mechanism and provide new insights into therapeutics for neurodegenerative conditions especially in early stage.

## Materials and methods

### Animals

Animal feeding and experiments were authorized by the Institutional Animal Research Committee of Huazhong University of Science and Technology and in conformity to the Guide for the Care and Use of Laboratory Animals (National Institute of Health, Bethesda, MD, USA). Cx3cr1-Cre^ERT2^, Raptor^F/F^ and Sirt1^F/F^ mice were purchased from Jackson Labs. The sex of subjects in this study was male.

### Reagents and antibodies

Corn oil was purchased from Maclin Biochemical (Shanghai, China, #C11808030). Resveratrol powder was obtained from Aladdin (Shanghai, China). Recombinant murine IFN-γ, EX527, resveratrol solution, tamoxifen, penicillin/streptomycin, cell lysis buffer for Western and BCA Protein Assay Reagent were bought from Beyotime (Shanghai, China). Rapamycin and NS1-IN-1 were purchased from MedChemExpress (Shanghai, China, HY-10219). l-Leucine was obtained from BioFroxx (Guangzhou, Guangdong, China). Lipopolysaccharides, Latex beads, amine-modified polystyrene (fluorescent red), Earle’s balanced salt solution (EBSS), papain and DNase were bought from Sigma Aldrich (Shanghai, China). DMEM/F12, HBSS with 0.25% trypsin, Proteases Inhibitor PMSF and phosphatase inhibitors were purchased from Boster (Wuhan, Hubei, China). SuperMix for qPCR (gDNA digester plus) Hifair III 1st Strand cDNA Synthesis, Hieff qPCR SYBR Green Master Mix and DMSO were obtained from Yeasen (Shanghai, China). WesternBright Peroxide and WesternBright ECL were bought from Advansta (Beijing, China). Poly-d-lysine coated flasks and Lipofectamine™ 3000 Transfection Reagent were obtained from Thermo Fisher Scientific (Waltham, USA). The following antibodies were used in immunofluorescence: anti-RBPMS antibody (ab152101; Abcam; 1:500); anti-β3-tubulin antibody (ab6161; Abcam; 1:1000); anti-synaptophysin antibody (ab32127; Abcam; 1:200); anti-Psd95 antibody (ab2723; Abcam; 1:200); anti-Iba1 antibody (ab5076 and ab178846; Abcam; 1:200); anti-CD68 antibody (ab955; Abcam; 1:50); DAPI (ab228549; Abcam; 1:1000); anti-TSC2 antibody (#4308; cell signaling technology; 1:800); anti-HA-Tag antibody (#sc-7392; Santa Cruz; 1:100). The following antibodies were used in western blot: anti-TSC2 antibody (#4308; cell signaling technology; 1:1000); anti-HA-Tag antibody (#sc-7392; Santa Cruz; 1:100); anti-Sirt1 antibody (ab189494; Abcam; 1:1000); anti-phospho-tuberin/TSC2 (Ser1387) antibody (#5584; cell signaling technology; 1:1000); anti-FLAG-Tag antibody (#8146; cell signaling technology; 1:1000); anti-p70 S6 kinase antibody (#2708; cell signaling technology; 1:1000); anti-phospho-p70 S6 kinase antibody (#9234; cell signaling technology; 1:1000). The following antibodies were used in immunoprecipitation (IP): anti-HA-Tag antibody (#sc-7392; Santa Cruz; 1:50); anti-Sirt1 antibody (ab189494; Abcam; 1:30); anti-FLAG-Tag antibody (#8146; cell signaling technology; 1:50); anti-TSC2 antibody (#4308; cell signaling technology; 1:50).

### Generation of microglial Raptor deficiency mice

Cx3Cr1-Cre^ERT2^/Raptor^F/F^ mice were obtained by crossing Raptor^F/F^ mice with Cx3cr1-Cre^ERT2^ mice and treated with tamoxifen before experiments. Tamoxifen is dissolved in corn oil at a concentration of 20 mg/ml by shaking overnight at 37 °C. For adult mice, a standard dose of 100 µl tamoxifen/corn oil solution is effective for inducing recombination. Administration of tamoxifen via intraperitoneal injection was started 9 days before ONC once every 24 h for a total of 5 consecutive days and continued every other day until the mice were killed.

### Animal model of optic nerve crush (ONC)

Mice aged 6–8 weeks old were anesthetized with sodium pentobarbital and the dose was 20 mg/kg. To expose optic nerve, tissues around optic nerve were dissected carefully. Then, ONC was performed at 5 mm behind the globe with N5 self-closing forceps and crush time were 10 s. In the histological experiments, only one eye of the mouse underwent ONC operation, while the contralateral eye served as a sham control. In visual function analysis, both eyes of each mouse were given ONC operations and the mouse as a whole was treated as an independent subject. Finally, mice were recovered at 37 °C on a warming pad before returning to cages.

### Cell culture and transfection

Immortalized murine microglial cell line BV2 and HEK-293T cell line, purchased from Procell (Wuhan, China), were cultured in Dulbecco’s modified Eagle’s medium (DMEM) supplemented with 10% Fetal Bovine Serum (FBS), 100 U/ml penicillin and 100 mg/ml streptomycin, and maintained at 37 °C and 5% CO_2_. HEK-293T and BV2 cells were transfected with Flag-TSC2 plasmid/vehicle and HA-Sirt1 plasmid/vehicle using Lipofectamine 3000 according to the manufacturer’s protocol. Flag-TSC2 plasmid and HA-Sirt1 plasmid were constructed at Tsingke biological technology.

Primary microglia cells were isolated from the retinas of neonatal mice as previously described [28, 29]. In brief, mixed retinal glial cultures were acquired via digesting isolated retinas by using papain containing 180 units/ml DNase at 38 °C for 20 min, followed by centrifuged. Then, resuspend retinas cells with DMEM/F12 (containing 10% FBS, 100 U/ml penicillin, and 100 mg/ml penicillin/streptomycin), and culture resuspended retina cells until reaching confluency. Finally, shake flasks in orbital shaker (200 rpm, 6 h) to collect detached microglia.

### Cell slide

Put coverslip into 12-well plate (1 coverslip per well) and seed BV2 cells on it. Add fresh and pre-warmed cell culture medium at 2 h post-seeding.

### Tissue preparation

Eyes and optic nerves were harvested from animals after killing and soaked in 4% paraformaldehyde (PFA).

For paraffin section, eyes were paraffin embedded for acquiring 4-μm sections after 24 h PFA-fixation at 4 °C and section was parallel to axis oculi. Eye sections containing optic nerve head were used for further analyzing. Before performing immunofluorescence staining, eye sections were dewaxed in environmentally safe clearing agent and gradient alcohol, washed with ddH_2_O and then soaked in trisodium citrate solution (pH = 6.0) for microwave antigen retrieval in 20 min.

For frozen section, after 24-h fixation in 4% PFA at 4 °C, optic nerves were cryoprotected in 30% sucrose for 24 h, followed by being frozen in OCT on the freezing element of a Leica CM1950 cryostat microtome to acquire 6-μm sections. Optic nerve sections were permeabilized with 0.3% Triton X-100 for 15 min and then washed 3 times using PBS for 5 min before immunofluorescence.

For flattening of mice retina, eyes were fixed in 4% PFA for 1 h at room temperature (RT). Then, eyes were cut through the sclera just posterior to the ciliary body and lens were removed. To separate retinas easily, eyecups were fixed in 4% PFA at RT for another 45 min. And 4 cuts were made toward optic disc to make the retinas lay flat. Retina tissues were permeabilized in − 20 °C methyl alcohol 30 min, washed with PBS 3 times, and then soaked in 0.3% Triton X-100 for 1 h prior to immunofluorescence.

### Immunofluorescence

After tissue preparation, tissue sections and retinas were blocked in donkey serum for 1 h and 12 h, respectively. For tissue sections, they were incubated in diluted primary antibodies at 4 °C overnight, followed by incubation in diluted secondary antibodies at room temperature for 2 h. The images acquisition of optic nerve and retina sections were processed under a fluorescence microscope (OLYMPUS BX51) at 40 × magnification. As for retinal section image analyzation, 5 pictures were acquired per sample and the mean data of the 5 pictures were used as sample data.

For retinal flat mounts, they were incubated in diluted primary antibodies at 4 °C for 72 h, followed by incubation in diluted secondary antibodies at 4 °C for 5 h. To visualize microglia and synapses in the inner retinal layer, the pictures of inner plexiform layer of retinal middle region (0.8 mm from the optic disc) were captured by a confocal microscope (Leica SP8). Pearson’s correlation coefficient was used to evaluate microglial phagocytosis to synapse in IPL, and 5 pictures (per sample) were acquired for analyzation.

### Reagents administration

#### In vivo experiment

Resveratrol (Res) powder was dissolved in corn oil at a concentration of 10 mg/ml. Mice were intragastrically administrated with resveratrol solution once a day and the dosage were 100 mg/kg. l-Leucine was dissolved in ddH_2_O at a concentration of 50 mM. l-Leucine solution was injected into vitreous body and the administration of l-leucine was performed on 2 days before and post-ONC, respectively.

#### In vitro experiment

For EX527 treatment, cells were treated with EX527 for 2 h before lipopolysaccharides (LPS) and IFN-γ treatment and the final concentration was 500 nM/ml. For Res treatment, solution was added to cell cultures (final concentration: 1 μM/ml) before LPS and IFN-γ treatment and the incubation time was 6 h. As for rapamycin, cells were treated with rapamycin for 2 h prior to LPS and IFN-γ treatment and the final drug concentration was 37.5 nM/ml. After pre-treated with above drugs, cells were treated with LPS and IFN-γ for 12 h and the final concentration were 1.25 μg/ml and 75 ng/ml.

### Wound-healing assay

BV2 cells were first seeded in 6-well plate, and the wound-healing assay was performed 24 h later. A scratch was applied on the cell monolayer via using 10-μl plastic pipette tip. Photomicrographs of the scratch area were taken at 0 h and 24 h post-operation. Five pictures were taken per well and the number of invading cells were counted by using Fiji. The mean of invading-cell number of 5 photos from one well was calculated as one sample data.

### Microglia phagocytosis assays

BV2 cells are seeded in the same way as described in “Cell slide”. Coverslips are put into a 12-well plate (1 coverslip per well) and the BV2 cells are seeded on the coverslips. Fresh and pre-warmed cell culture medium are added at 2 h post-seeding. 24 h were required for BV2 cells to recover, after which the phagocytosis assay was performed. Aqueous red fluorescent latex beads were pre-opsonized in FBS for 1 h at 37 °C and the ratio of beads to FBS was 1:5. Beads-containing FBS was diluted with DMEM to reach the final concentrations for beads and FBS in DMEM of 0.01% (v/v) and 0.05% (v/v), respectively. BV2 cell culture media was replaced with beads-containing DMEM. After one-hour incubation at 37 °C, cultures were washed with ice-cold PBS 5 times and then fixed with 4% PFA for 15 min. BV2 cells and fluorescent latex beads were visualized with ordinary light channel and red channel. The number of cells which phagocytized beads was counted via Fiji.

### Amino acid starvation and l–Leucine stimulation

For amino acid starvation experiments, BV2 cells were washed with PBS for 3 times and incubated for 2 h at 37 °C in Earle’s balanced salt solution (Starvation media). After starvation, l-leucine solution was added into culture medium (final concentration of l-Leucine was 10 mM) and cell cultures were incubated at 37 °C for 1 h.

### Immunoblot and immunoprecipitation

Cells were treated with RIPA Lysis which was supplemented with protease and phosphatase inhibitor at designed time-points. Protein concentration was quantified via using BCA Protein Assay Reagent. When performing immunoprecipitation, same amounts of whole-cell lysate were incubated with the primary antibodies (0.5–2 μg) overnight at 4 °C, protein A/G sepharose beads were added into the incubation tubes, and then the mixture was incubated at 4 °C with gentle shaking for 3 h. Finally, the precipitated complexes were washed five times with RIPA buffer, mixed with loading buffer and boiled at 100 °C for 5 min. For Western Blot analysis, protein samples were separated by SDS-PAGE, transferred onto PVDF membranes, and finally visualized via an ECL chemiluminescence detection kit on a CLiNX ChemiScope Chemiluminescence Imaging System. Fiji was used to perform densitometric analysis on the immunoblots.

### Plasmid constructs and transfection

Plasmids were constructed as we previously described [30, 31]. Briefly, the full-length DNA segment SIRT1 coding sequence was amplified by PCR, and cloned into HA-tagged pcDNA 3.0 (HA-SIRT1). For Flag-TSC2 plasmid, PCR was applied to amplify the full-length cDNA of human TSC2, and cloned into indicated vectors including pFlag-CMV2, respectively. For site-directed mutagenesis, the subsequent mutants Flag-TSC2-K1473R, Flag-TSC2-K1165R (lysine-to-arginine), and SIRT1-H363Y (histidine-to-tyrosine) were constructed using homologous recombination via Trelief SoSoo Cloning Kit Ver.2 (TSINGKE, Beijing, China) reference to product specification. All constructs were confirmed by DNA sequencing analysis (performed by Sangon Biotechnology, Shanghai, China). The recombinant plasmids were transfected into cells using Lipofectamine 3000 (Invitrogen, NY, USA) when the cells were 80–90% confluent, following the manufacturer’s instructions.

### RNA isolation and real-time quantitative PCR analysis

Total RNA was isolated from BV2 cell cultures via using RNAiso plus and its concentration was quantified by NanoDrop 2000 (Thermo Fisher Scientific). RNA was reverse transcribed into cDNA using Hifair^®^III 1st Strand cDNA Synthesis according to the manufacturer’s protocol. Quantitative PCR was performed on Quantagene q225 (Kubo Tech Co., Ltd.), using Hieff^®^qPCR SYBR Green Master Mix. The primer sequences were as follows:

*IL-1β*: Forward TGCCACCTTTTGACAGTGATG, Reverse CCCAGGTCAAAGGTTTGGAA.

*iNOS*: Forward GCTTGTCTCTGGGTCCTCTG, Reverse CTCACTGGGACAGCACAGAA.

*TNF-α*: Forward ACGGCATGGATCTCAAAGAC, Reverse AGATAGCAAATCGGCTGACG.

*IL-6*: Forward GCTGGAGTCACAGAAGGAGTGGC, Reverse GGCATAACGCACTAGGTTTG.

CCGA. *β-actin*: Forward ATCTTCCGCCTTAATACT, Reverse GCCTTCATACATCAAGTT.

All samples were run in triplicate with blank controls. The relative expression of target genes was calculated by the 2^−ΔΔCT^ method with normalization against β-actin level.

### Visual function analysis

Mice were treated with oral Res or vehicle before ONC of both eyes. Each mouse was viewed as an independent subject, since the device was unable to distinguish the difference between left and right eyes in visual function. Visual function analysis was performed on day 5 and day 21 post-crush. For the pupillary light reflex test (PLR), mice were first dark-adapted for at least 1 h to allow maximal pupil dilation. Dark-adapted mice were hand-restrained, and the eyes were recorded by a camera under a 470-nm LED light source (30 lx). The percentage change in pupil size at 30 s after the initiation of the light stimulus relative to the fully dilated pupil size before the light stimulation was calculated by ImageJ and used as the percentage of pupil constriction. For the dark light preference test, the apparatus used consisted of a box divided into a small (one-third) dark chamber and a large (two-thirds) illuminated chamber (600 lx) connected by a small opening. Mice were allowed between the two compartments to move freely for 10 min. The time spent in each compartment, time ratio and traveling trajectories were automatically analyzed (SuperMaze, XINRUN) based on the behavior recorded by the camera of Light–Dark Box System XR-XB120 (XINRUN). For the optomotor response test, mice were placed and freely moving on a central, elevated platform surrounded by four screens displaying a moving vertical sinusoidal grating pattern. The spatial frequency began from 0.01 to 0.06 cycles per degree with constant rotation speed (12°/s) and 100% contrast to determine the spatial frequency threshold at which the mice still tracked the moving grid. Different testing frequencies occurred randomly and repeated 10 times within one test to reduce the occasional error. Observers were blinded to the group of mice when performing visual function analysis.

### Statistical analysis

Data shown represent three independent experiments at least. Significance levels for comparisons between groups were determined by two-tailed t tests, one-way ANOVA or Dunnett’s multiple comparisons test using GraphPad Prism (v. 9.3.1; GraphPad Software, La Jolla, CA, USA). Error bars represent mean ± S.E.M. For all tests, *P* values of < 0.05 were considered statistically significant.

## Results

### ONC induces synapse loss before RGC soma loss, accompanied by microglia overactivation

**Fig. 1:**
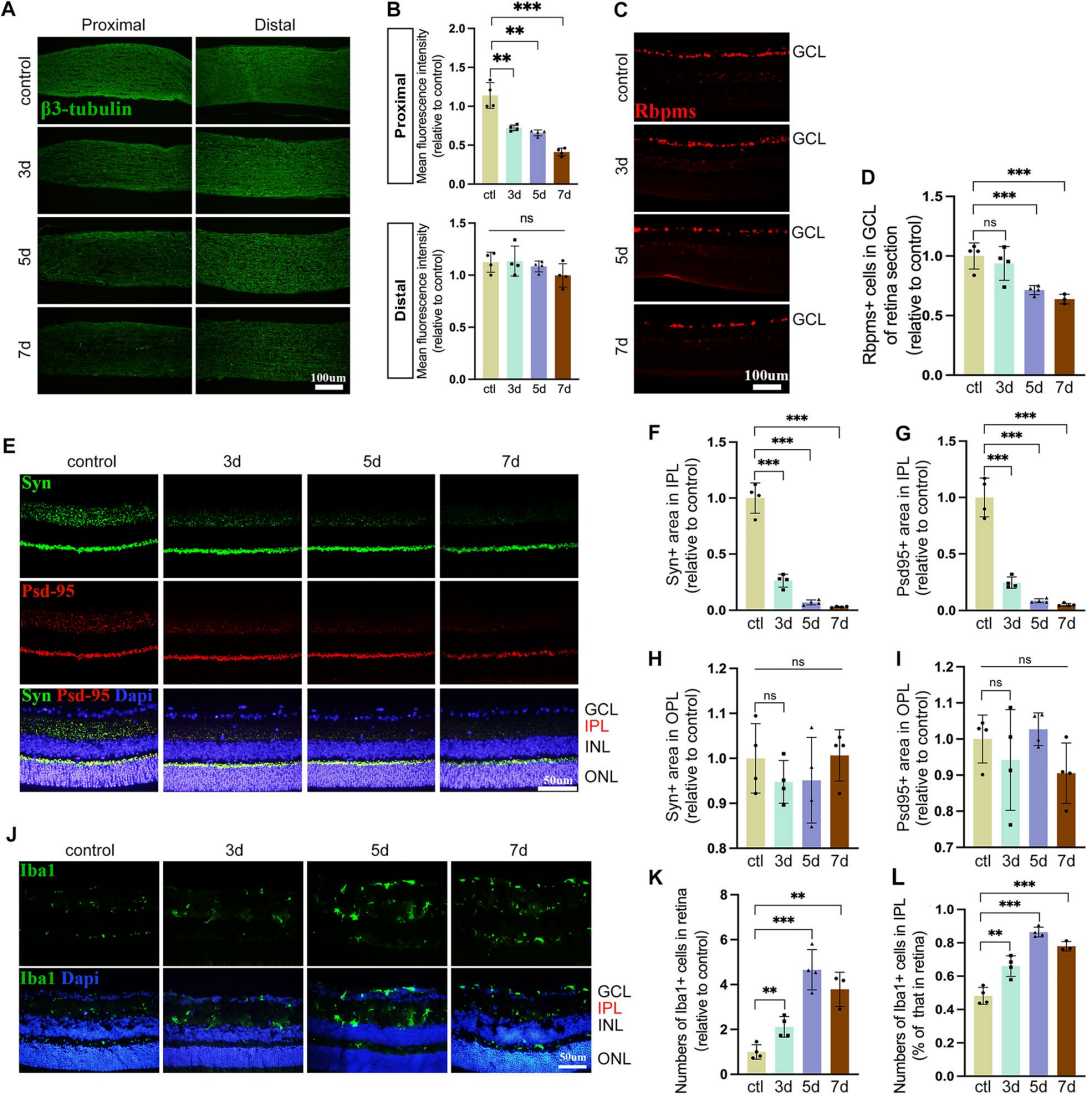
ONC induces RGC excitatory synapse injury before soma loss, accompanied by microglia overactivation. **A** Representative images of RGCs axon, stained by β3-tubulin, at proximal and distal site of the crush site of Control and 3d, 5d, and 7d post-ONC. The fluorescence intensity of β3-tubulin near injury site decreased continuously after ONC, while no significant change observed at the distal site. Scale bar, 100 μm. **B** Quantification of β3-tubulin fluorescence intensity in **A**. **P < 0.01, ***P < 0.001, ns versus Control group. *N* = 4. **C** Representative images of RGCs, detected by Rbpms (RGCs marker), of retina from Control and 3d, 5d, and 7d post-ONC. Scale bar, 100 μm. **D** Measurement of the number of Rbpms positive cells in GCL in **B**. ***P < 0.001, ns versus Control group. ns, no significance. *N* = 4. **E**–**I** Synapse loss in retinal IPL, detected by synapse marker (Syn and Psd95), started at 3d post injury, while no significant synapse loss was observed in OPL. Images of retinal synapse (**E**) and quantification of Syn and Psd95 positive area in IPL (**F**, **G**) and in OPL (**H**, **I**) are presented. Scale bar, 50 μm. ***P < 0.001, ns versus Control group. *N* = 4. **J**–**L** Microglia activation, assessed by Iba1 (microglial marker) occurred at 3d post-ONC, reached peak at 5d post-ONC and slightly decreased at 7d post injury. Shown are images of the distribution of microglia in retina from Control and 3d, 5d, and 7d post-ONC (**J**) and quantification of the number of Iba1 positive cells in retina (**K**) and IPL (**L**). Scale bar, 50 μm. **P < 0.01, ***P < 0.001, ns versus Control group. *N* = 4

After establishing ONC model, mice were killed at various intervals (day 3, 5, 7 after crush). Axonal damage was assessed by anti-β3 tubulin immunofluorescence. It displayed gradually developing axon damage after the crush both at the proximal and distal axon following the crush injury (Fig. 1A, B). We performed anti-Rbpms immunofluorescence staining in the retinas of mice to estimate the number of RGCs. It showed that the number of Rbpms-positive cells decreased significantly on day 5 and 7 after the crush, while there were still no significance changes on day 3 (Fig. 1C, D). To determine whether ONC is linked to synapse loss, retinal samples were evaluated for the expression of synaptic markers, synaptophysin and PSD-95, using immunofluorescence staining [32]. Three days following the ONC model, in crushed retinas, a significant drop in synaptophysin and PSD-95 was observed in the inner plexiform layer (IPL), while there was no discernible trend in the outer plexiform layer (OPL) (Fig. 1E–I). Then we looked into whether microglia were involved in ONC-induced alterations in the retina. Immunofluorescence demonstrated that the number of Iba1-positive cell began to grow on day 3 after ONC, rose until it peaked at day 5 post-injury and then began to decline 2 days later in the retinas (Fig. 1J–L), which was consistent with neuroinflammation in the optic nerve [26]. Taken together, our data suggested that synapse loss and microglia activation began sooner than RGCs soma loss after ONC. Hence, we hypothesized that there might be a relation between microglial activation and retinal synapse as well as RGC soma loss post-injury. Given that the time point of the change in retina we captured, our subsequent research concentrated on synapse change and microglial activation during the early stage of ONC (3d post-ONC), and evaluated RGC soma loss at 5d post-ONC.

### SIRT1 inhibits microglial activation to protect RGCs and synapse from injury

**Fig. 2:**
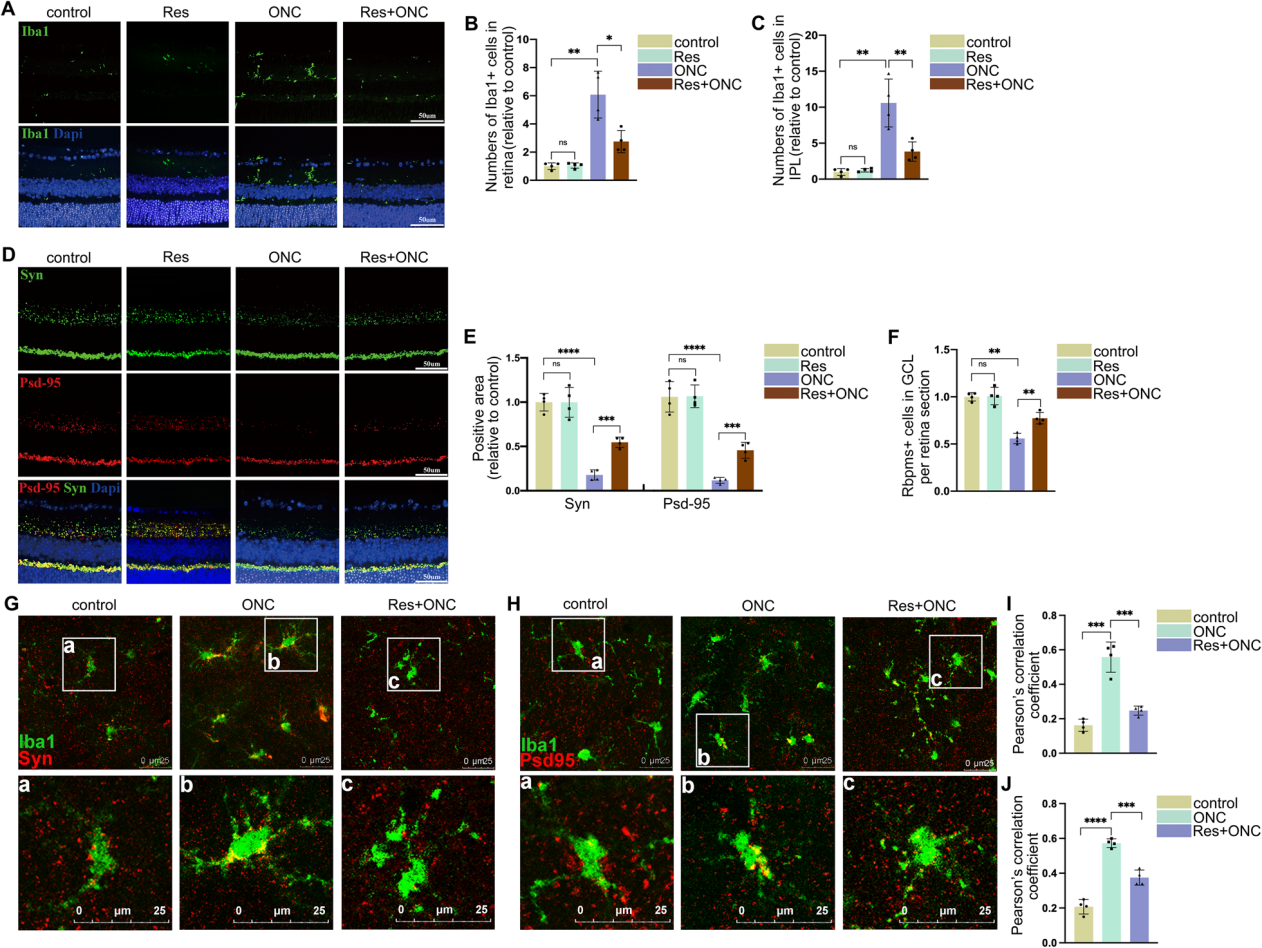
SIRT1 inhibits microglial activation to protect RGCs and synapse from injury. (**A**, **D**, **F**) Sirt1 activation, triggered by Res, attenuated microglial activation and synapse loss induced by ONC, and protected RGCs from loss post injury. Representative images of microglial activation (**A**) and synapse (**D**) of retina from mice treated with or without ONC or Res, as well as the number of Rbpms positive cell in GCL (**F**) are presented. Scale bar, 50 μm. For (**F**): ns, **P < 0.01 versus control mice; **P < 0.01 versus ONC mice. *N* = 4. (**B**, **C**, **E**) Quantitative measurement of the number of Iba1 positive cells in retina (**B**) and IPL (**C**) of (**A**), and quantification of Syn and Psd95 positive area (**E**) in (**D**). For (**B**): *P < 0.05 versus ONC mice; ns, **P < 0.01 versus control mice. *N* = 4. For (**C**): ns, **P < 0.01 versus control mice; **P < 0.01 versus ONC mice. *N* = 4. For (**E**): ns, ****P < 0.0001 versus control mice; ***P < 0.001 versus ONC mice. *N* = 4. **G**–**J** Microglia cells phagocytose synapse in retinal IPL after ONC, and Sirt1 could inhibit this process to attenuate injury induced synapse loss. Representative images of Microglial phagocytosis to synapse in IPL of mice without injury and mice with or without Res treatment post-ONC (**G**, **H**) and quantification analysis of colocalization of Iba1 with Syn (**I**) or Psd95 (**J**) in (**G**) and (**H**) are presented. ***P < 0.001 versus mice treated with ONC. *N* = 4

To investigate the role of SIRT1 in *vivo*, resveratrol gavage was applied. Histone 3 lysine 9 (H3K9) is the downstream target of SIRT1, and SIRT1 activity could be measured by detecting the level of acetylation of H3K9 (ac-H3K9) [33–35]. As shown in Additional file 1: Fig. S1F, by using paved retinas, we detected an increased expression of ac-H3K9, and elevated colocalization of anti-iba1, anti-ac-H3K9 and Dapi in inner plexiform layer of ONC mice compared to control, while resveratrol treatment could decrease the expression of ac-H3K9 and the colocalization. This result implies that resveratrol gavage can effectively increase the activity of SIRT1. Immunostaining for Iba1 did not reveal overt differences in microglial density when resveratrol was used without ONC. But there was a significant drop in the number of ﻿microglia in resveratrol-treated mice when compared with ONC-treated mice (Fig. 2A–C). Most significantly, immunofluorescence demonstrated that resveratrol could attenuate synapses loss in the IPL layer after ONC injury (Fig. 2D, E). Additionally, resveratrol treatment could also reduce RGCs loss (Fig. 2F). Hence, SIRT1 activation inhibits microglial activation without influencing microglial survival or proliferation, and exerts protection effect on RGCs and synapse.

Activated microglia secreted inflammation factors which were harmful to neuron survival [36]. As shown in Additional file 1: Fig. S1A, SIRT1 activation could significantly reduce the expression level of inflammation factors in microglia triggered by LPS and IFN-γ. Given that microglial M1 phenotype is the proinflammatory phenotype, we detected the M1 marker iNOS in vivo [37]. ONC resulted in activation of M1 phenotype microglia in IPL, while SIRT1 activation by Res could alleviate this process (Additional file 1: Fig. S1G). As microglia migrate to injury site and phagocyte debris and neuron when injury occurred, we evaluated whether SIRT1 activation could exert influence on microglial migration and phagocytosis capacity. Once activated by LPS and IFN-γ, the number of microglia cells migrating to scratch increased compared to Vehicle group. But microglial SIRT1 activation induced by resveratrol attenuated microglial migration (Additional file 1: Fig. S1B, C). To further investigate microglial phagocytosis capacity, we performed beads-phagocytosis assay. SIRT1 activation dramatically decreased the amount of fluorescent beads that activated microglia phagocytosed compared to the control group (Additional file 1: Fig. S1D, E). To clarify how microglia mediate synapse loss after ONC, confocal microscopy was used to detect microglia and synapse in IPL. As shown in Fig. 2G–I, whole retina mounts showed a considerable increase in the colocalization between synaptic markers (Syn or Psd95) and Iba1 in Iba1 positive areas after ONC, while resveratrol restored this effect.

Taken together, the findings revealed that following ONC, activated microglia phagocytize synapse in IPL. By preventing microglial migration capacity, phagocytosis of synapses and suppressing microglial proinflammatory molecules expression, SIRT1 exhibited neuron protective effects.

### SIRT1 mediated deacetylation of TSC2 at lysine residues 1473

**Fig. 3:**
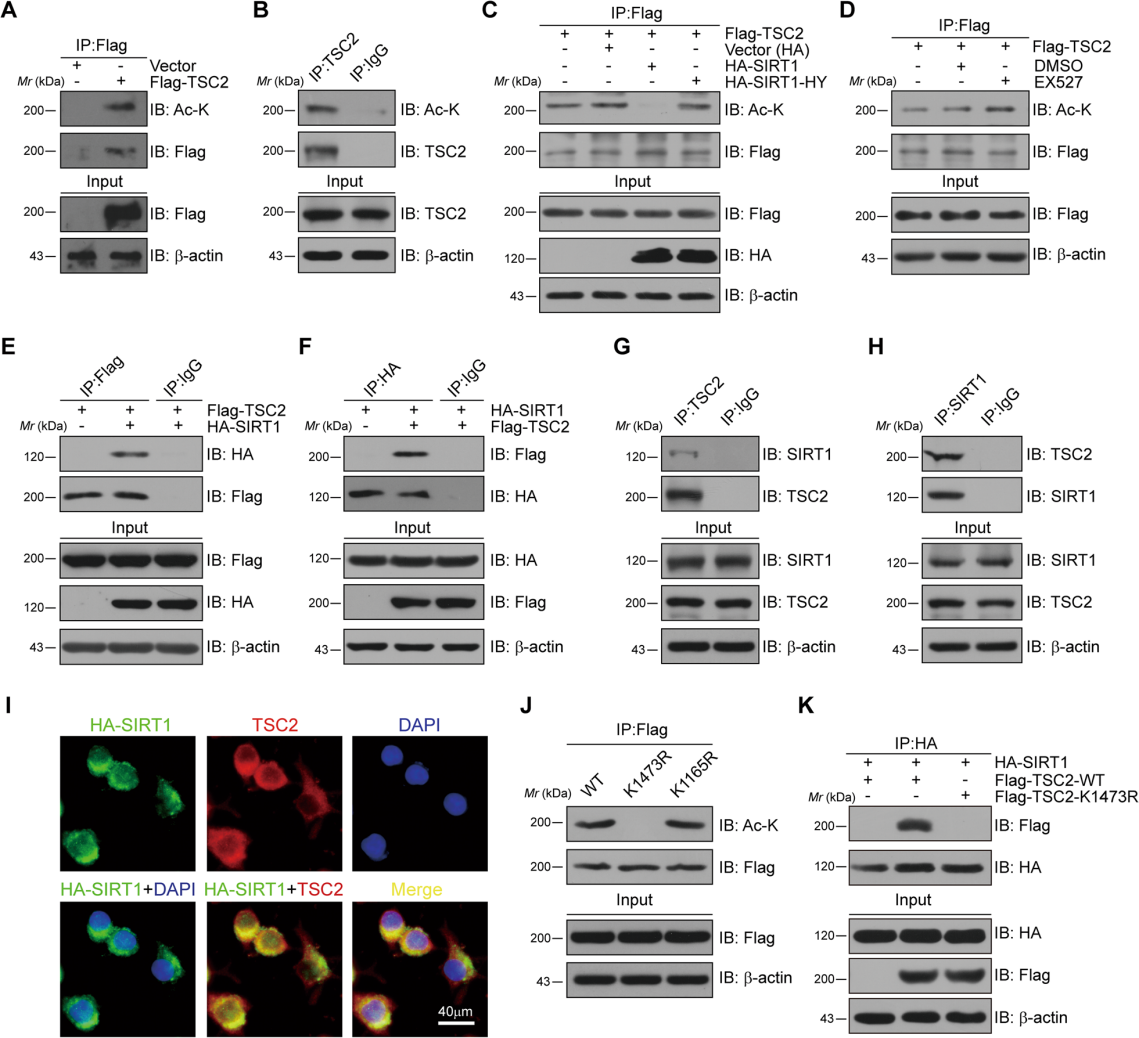
SIRT1 mediates the deacetylation of TSC2 at lysine residues 1473. **A** Exogenous TSC2 was targeted by acetylation in HEK293 cells. Shown is immunoblot (IB) analysis of whole-cell lysates (input) and anti-Flag immunoprecipitates derived from HEK293 cells transfected with the indicated constructs. **B** Endogenous TSC2 was targeted by acetylation in BV2 cells. Shown is IB analysis of input and anti-TSC2 immunoprecipitates derived from BV2 microglia cells. IgG IP served as control. **C** Sirt1 promoted TSC2 deacetylation in cells. Shown is IB analysis of input and anti-Flag immunoprecipitates derived from HEK293 cells transfected with Flag-TSC2 along with hemagglutinin (HA)-tagged constructs. HA-SIRT1-HY, catalytically inactive SIRT1. **D** Treatment with EX527, Sirt1 specific inhibitor, could reduce TSC2 acetylation. Shown is IB analysis of input and anti-Flag immunoprecipitates derived from HEK293 cells transfected with Flag-TSC2 along with no another treatment, DMSO treatment and EX527 treatment, respectively. **E**, **F** Exogenous interaction between TSC2 and Sirt1. Shown is IB analysis of input and anti-Flag (**E**) or anti HA (**F**) immunoprecipitates derived from HEK293 cells transfected with Flag-TSC2 along with hemagglutinin (HA)-tagged constructs or transfected with HA-Sirt1 along with Flag-tagged constructs. IgG IP served as control. **G**, **H** Endogenous interaction between TSC2 and Sirt1. Shown is IB analysis of input and anti-TSC2 (**G**) or anti-Sirt1 (**H**) immunoprecipitates derived from BV2 cells. IgG IP served as control. **I** Representative immunofluorescence images showing colocalization of HA-Sirt1 (green) and TSC2 (red) in BV2 cells. Scale bar, 40 μm. **J** Acetylation-deficient K1473R significantly diminished TSC2 acetylation. Shown is IB analysis of input and anti-Flag immunoprecipitates derived from HEK293 cells transfected with Flag-TSC2. **K** Acetylation-deficient TSC2 K1473R mutant markedly diminished the interaction between Sirt1 and TSC2. Shown is IB analysis of input and anti-HA immunoprecipitates derived from HEK293 cells transfected with HA-Sirt1 along with Flag-tagged constructs

First, we investigated whether TSC2 could be modified by acetylation in microglia. To address this question, Flag-tagged TSC2 plasmids were transfected into human embryonic kidney (HEK) 293T cells. The co-immunoprecipitation (Co-IP) assay results demonstrated that ectopically expressed TSC2 was able to be modified by acetylation (Fig. 3A). Further research also validated that endogenous TSC2 can be acetylated in BV2 microglial cells (Fig. 3B). Next, we investigated whether SIRT1 can mediate the deacetylation of TSC2. Flag-tagged TSC2 along with HA-tagged SIRT1 or HA-tagged SIRT1-HY, were transfected into HEK293T cells. When histidine (H) at position 363 of SIRT1 was mutated into tyrosine (Y) (abbreviated as SIRT1-HY), the deacetylase activity of SIRT1 would be lost [38]. The co-immunoprecipitation (Co-IP) assay results showed that HA-SIRT1-WT markedly decreased the acetylation level of TSC2, while HA-SIRT1-HY mutant had no impact (Fig. 3C). Meanwhile, EX527, a selective inhibitor of SIRT1, appeared to be able to raise the acetylation level of TSC2 in whole cell lysates from HEK293T cells (Fig. 3D).

To further determine whether TSC2 is a putative substrate of SIRT1, we subsequently explored the interaction of TSC2 and SIRT1. HEK293T cells were transfected with HA-SIRT1 and Flag-TSC2 expression plasmids. We proved that ectopically expressed SIRT1 could interact with TSC2 (Fig. 3E). Reverse co-IP assays also supported this conclusion (Fig. 3F). Furthermore, it was demonstrated that endogenous SIRT1 could bind with TSC2 in BV2 microglial cells. The results revealed that endogenous SIRT1 can bind with TSC2 (Fig. 3G, H). These findings were further confirmed by immunofluorescence staining in BV2 microglial cells (Fig. 3I). Then, the acetylation sites of TSC2 were predicted by two different bioinformatical software algorithms, AESB (http://bioinfo.bjmu.edu.cn/huac/) and PHOSIDA (www.phosida.com/). K1473 and K1165 were rated as the top two by reliability. Flag-tagged TSC2-K1473R or K1165R plasmids was constructed and transfected into HEK293T cells. As shown in Fig. 3J, K1473R mutant exhibited lower acetylation levels, demonstrating that K1473 might be the primary acetylation site for TSC2. Moreover, we discovered that K1473R mutant significantly reduced TSC2’s interaction with SIRT1 (Fig. 3K). These data findings collectively suggest that TSC2 might undergo acetylation modification and SIRT1 was responsible for deacetylating TSC2 at lysine 1473.

### SIRT1-mediated deacetylation of TSC2 decreases its phosphorylation at tyrosine 1462

**Fig. 4:**
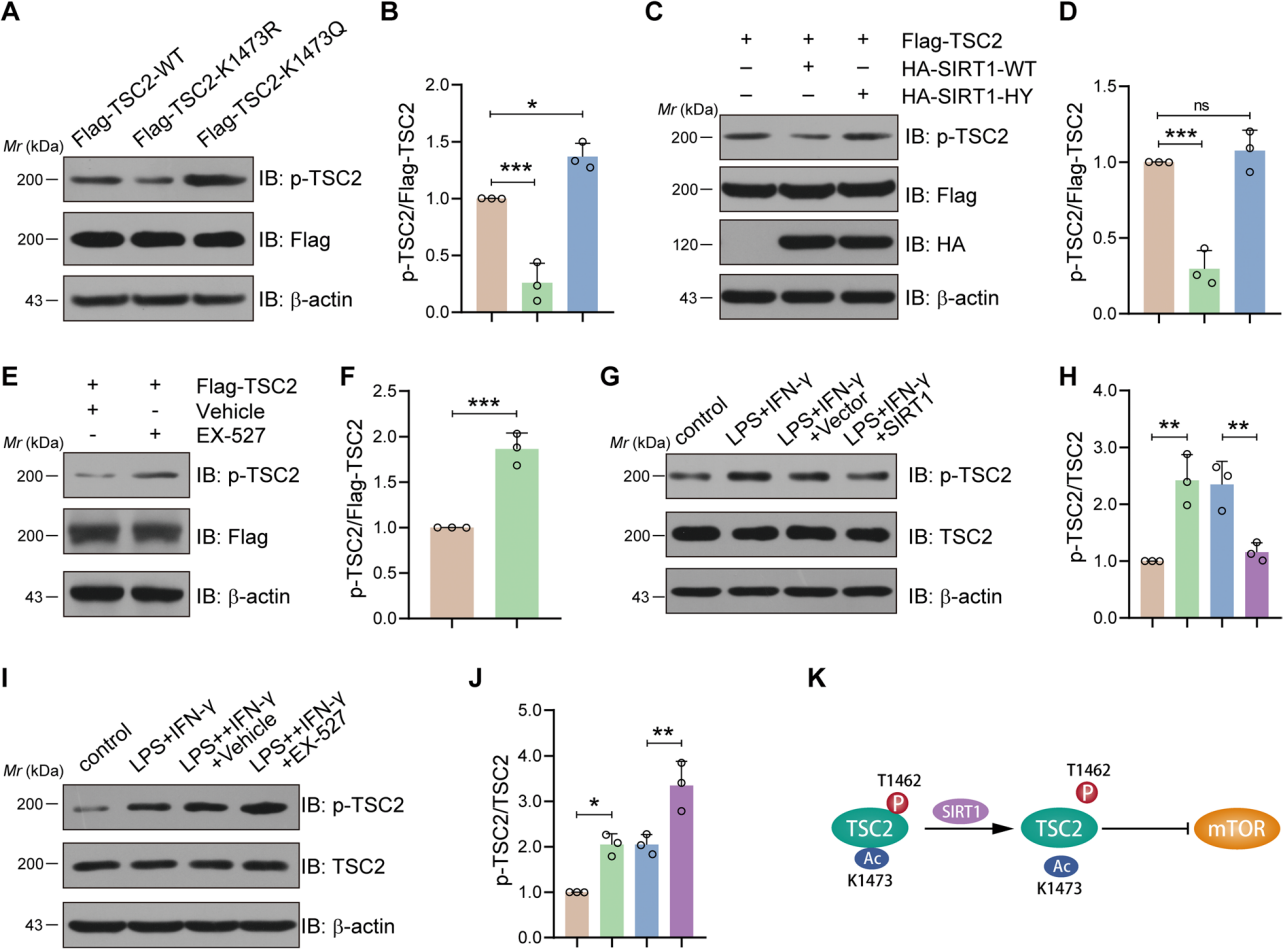
SIRT1-mediated TSC2 deacetylation decreases TSC2 phosphorylation at tyrosine 1462. **A** TSC2 acetylation was related to its phosphorylation. When compared with wildtype TSC2, phosphorylation level increased in acetylation-mimetic TSC2 K1473Q mutant while decreased in acetylation-deficient TSC2 K1473R mutant. Shown is IB analysis of whole-cell lysates derived from HEK293 cells transfected with Flag-tagged constructs. **B** Ratio of the p-TSC2 band intensities to Flag band intensities in **A**. *P < 0.05; **P < 0.01. *N* = 3. **C** Overexpression of Sirt1 reduced TSC2 phosphorylation. Shown is IB analysis of whole-cell lysates derived from HEK293 cells transfected with Flag-TSC2 along with HA-tagged constructs. **D** Ratio of the p-TSC2 band intensities to Flag band intensities in **C**. ***P < 0.001; ns, *P* > 0.05. *N* = 3. **E** Inhibition of Sirt1 activity via EX527 increased TSC2 phosphorylation level. Shown is IB analysis of whole-cell lysates derived from HEK293 cells transfected with Flag-TSC2 along with or without EX527 treatment. **F** Ratio of the p-TSC2 band intensities to Flag band intensities in **E**. ***P < 0.001. *N* = 3. **G** Sirt1 overexpression could attenuate TSC2 phosphorylation level induced by LPS plus IFN-γ treatment. Shown is IB analysis of whole-cell lysates derived from BV2 cells under different intervention. **H** Ratio of the p-TSC2 band intensities to Flag band intensities in **E**. **P < 0.01. *N* = 3. **I** EX527 induced inhibition of Sirt1 activity could further enhance TSC2 phosphorylation level induced by LPS plus IFN-γ treatment. Shown is IB analysis of whole-cell lysates derived from BV2 cells under different intervention. **J** Ratio of the p-TSC2 band intensities to Flag band intensities in **I**. *P < 0.05; **P < 0.01. *N* = 3. **K** Schematic drawing of the interaction of Sirt1, TSC2 and mTOR. SIRT1 deacetylated TSC2 at lysine residues 1473, further reduced its phosphorylation level of TSC2 at tyrosine 1462 and finally activated TSC2 to inhibit mTOR

Previous study had demonstrated that Akt-mediated phosphorylation of TSC2 at tyrosine 1462 are critical for tuberin–hamartin complexes formation and plays a critical inhibition role in mTOR signaling pathway activation [39–41]. To comprehend the mechanisms underlying SIRT1-mediated mTOR signaling pathway inhibition, we further investigated the interplay between TSC2 phosphorylation and acetylation. Frist, HEK293T cells were transfected with plasmids encoding Flag-TSC2-WT, Flag-TSC2-K1473R or Flag-TSC2-K1473Q (Q, mimics lysine acetylation), respectively. The immunoblot results revealed that, in contrast to TSC2-WT transfection, the phosphorylation of TSC2 at T1462 obviously decreased after K1473R, while increasing after K1473Q (Fig. 4A, B). Next, Flag-SIRT1-WT and Flag-SIRT1-HY were transfected into HEK293T cells. The immunoblot results suggested that SIRT1-WT dramatically decreased the phosphorylation level of TSC2, while the catalytic-deficient mutant SIRT5-HY had little impact (Fig. 4C, D). In addition, EX527 could significantly increase the phosphorylation level of TSC2 in BV2 microglial cells under normal conditions (Fig. 4E, F). More significantly, when BV2 microglial cells were treated with LPS and IFN-γ, the immunoblot results indicated that in contrast to the vector group, LPS plus IFN-γ significantly increased the phosphorylation level of TSC2, but SIRT1 overexpressing dramatically blocked the phosphorylation level of TSC2, as compared with the vector group. In contrast, we found that EX527 significantly enhanced the phosphorylation level of TSC2 induced by LPS and IFN-γ treatment in BV2 cells (Fig. 4G–J). Together, our findings suggest that TSC2's phosphorylation can be reduced by SIRT1-mediated deacetylation (Fig. 4K, mechanism's diagram).

To further investigate the role of TSC2 in mediating SIRT1 neuron protection effect, we applied TSC2 inhibitor, NS1-IN-1, to block the activity of TSC2. As shown in Additional file 1: Fig. S2A, B, once TSC2 activity blocked by NS1-IN-1, SIRT1 activation could not attenuate the increase of microglia cell number induced by ONC. Similarly, synapse loss increased when TSC2 was in low activity state, compared to Res group (Additional file 2: Fig. S2C, D). Hence, these findings suggest that SIRT1 exert neuron protection via modulating TSC2 activity.

### Microglial mTORC1 deficiency inhibits ONC-induced microglial activation

**Fig. 5:**
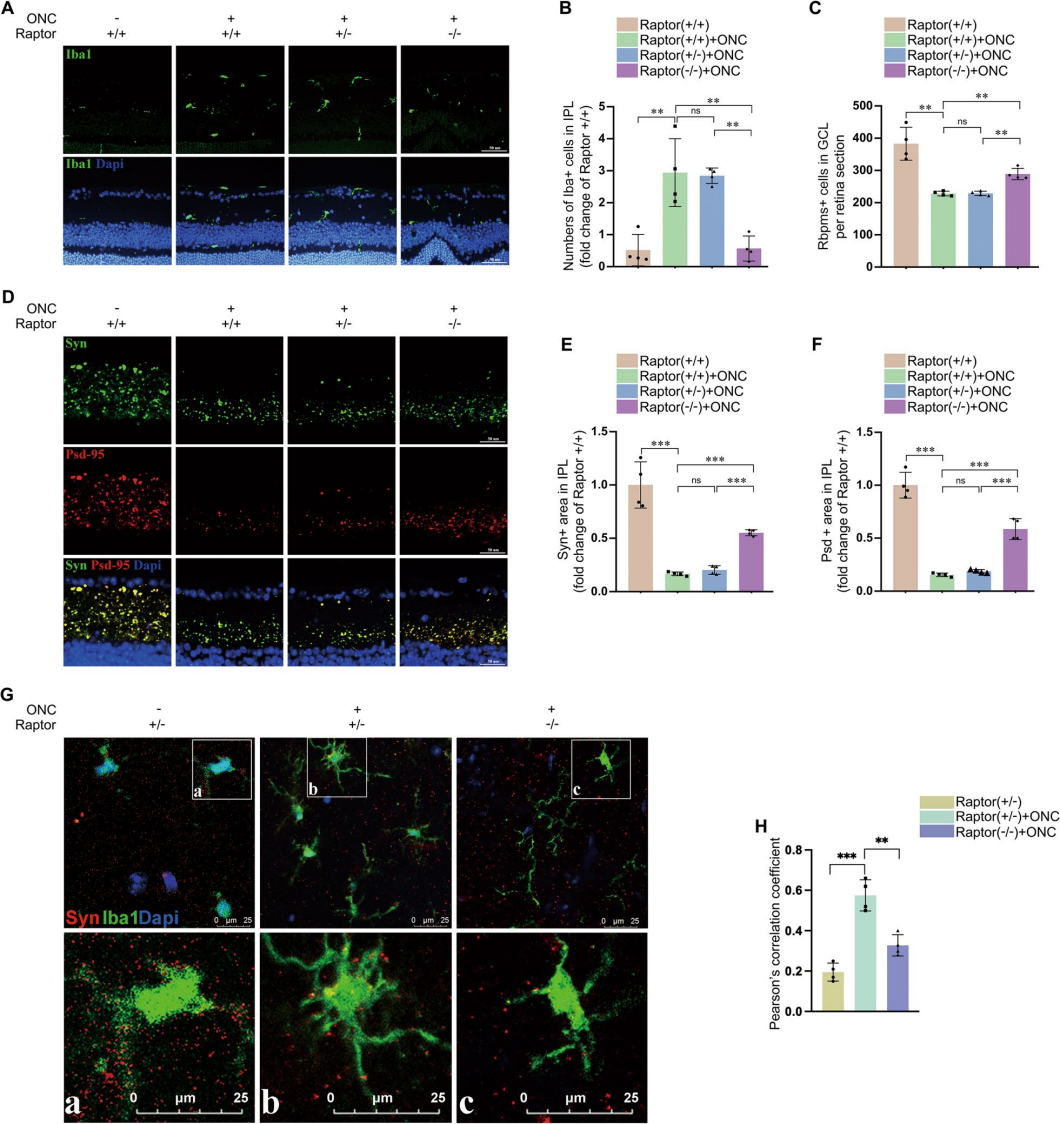
Microglial mTORC1 deficiency inhibits ONC-induced microglial activation. **A** Microglial mTORC1 deficiency exerted inhibition effect on microglial activation induced by ONC. Images of the distribution of microglia in retinas of Raptor (+/+), Raptor (±) and Raptor (−/−) mice with or without ONC are presented. **B** Quantification of the number of Iba1 positive cells in IPL of retinas in **A**. **P < 0.01, ns versus Raptor (+/+) mice with ONC; **P < 0.01 versus Raptor (±) mice with ONC. ns, no significance. *N* = 4. **C** Quantification of Rbpms positive cells in retinal GCL of Raptor (+/+), Raptor (±) and Raptor (−/−) mice with or without ONC. **P < 0.01, ns versus Raptor (+/+) mice with ONC; **P < 0.01 versus Raptor ( ±) mice with ONC. ns, no significance. *N* = 4. **D** Microglial mTORC1 deficiency protected against ONC-induced synapse loss in IPL. Shown is the representative images of synapse in retinal IPL of Raptor (+/+), Raptor (±) and Raptor (−/−) mice with or without ONC treatment. **E**, **F** Quantitative measurement of Syn and Psd95 positive area in **D**. ***P < 0.001, ns versus Raptor (+/+) mice with ONC; ***P < 0.001 versus Raptor (±) mice with ONC. ns, no significance. *N* = 4. **G** Microglial mTORC1 deficiency protects synapse from loss via attenuating microglial phagocytosis to synapse. Shown is the co-immunofluorescent staining images of Iba1 (green) with Syn (Red) in retinal IPL of Raptor (±) mice without ONC and Raptor (±) and Raptor (−/−) mice with ONC. **H** Quantification analysis of colocalization between Iba1 and Syn in **G**. **P < 0.01 versus Raptor (±) mice with ONC. *N* = 4

Mammalian target of rapamycin complex 1 (mTORC1) was reported to play a role in mediating of TLR5-mediated microglial activation [42]. Previous research showed that Sirt1 mediated cancer, liver disease and mitophagy via regulating mTORC1 [43–45]. We investigated how mTORC1 affected the activation of microglia. Raptor, a specific member of mTORC1, is involved in the formation and the recruitment process to lysosome of mTORC1 [46]. Microglial Raptor deficiency mice were obtained in the manner described in Material and methods, and the effectiveness of microglial Raptor knockout were evaluated (Additional file 3: Fig. S3A). Raptor deficiency in microglia significantly relieved the number of microglia in IPL induced by ONC (Fig. 5A, B). Quantification analysis showed that microglial *Raptor* deficiency in mice retina had a protective impact on RGC synapse. Less anti-Syn and anti-PSD-95 loss was confirmed by immunofluorescence of retina sections (Fig. 5D, F). Results show that more Rbpms-positive cells survived post-ONC in microglial *Raptor* knockout mice (Fig. 5C). As for further evaluating the effect of mTORC1 on microglial inflammation factor expression, migration, and phagocytosis, in vivo and in vitro experiments were performed. In vitro, mTORC1 activity were blocked by rapamycin, the specific inhibitor of mTORC1 [42]. As shown in Additional file 3: Fig. S3B, inhibiting mTORC1 activity reduced microglial inflammation factor expression compared with LPS + IFN-γ treatment group. And rapamycin could blunt the increased microglial migration induced by LPS + IFN-γ treatment (Additional file 3: Fig. S3C, D). To evaluate phagocytosis activity, immunofluorescence of CD68, a phagocytic marker for microglial, was performed in BV2 cells to assess phagocytosis activity [47]. LPS + IFN-γ treatment increased the expression level of CD68, which could be attenuated by rapamycin administration (Additional file 3: Fig. S3E, F). And we also evaluate the microglia/macrophage phagocytosis in vivo by using confocal microscopy. After ONC injury, microglia went into an active state with greater phagocytosis capability, while Raptor deficiency in microglia inhibited the phagocytosis to synapse (Fig. 5G, H). These results suggested that inhibiting mTORC1 might downregulate microglial activation and exhibit neuron protection effect.

### SIRT1–mTORC1 signal pathway is engaged in microglial activation and phagocytose of retinal synapse, and SIRT1 activation partially promotes visual function recovery after injury

**Fig. 6:**
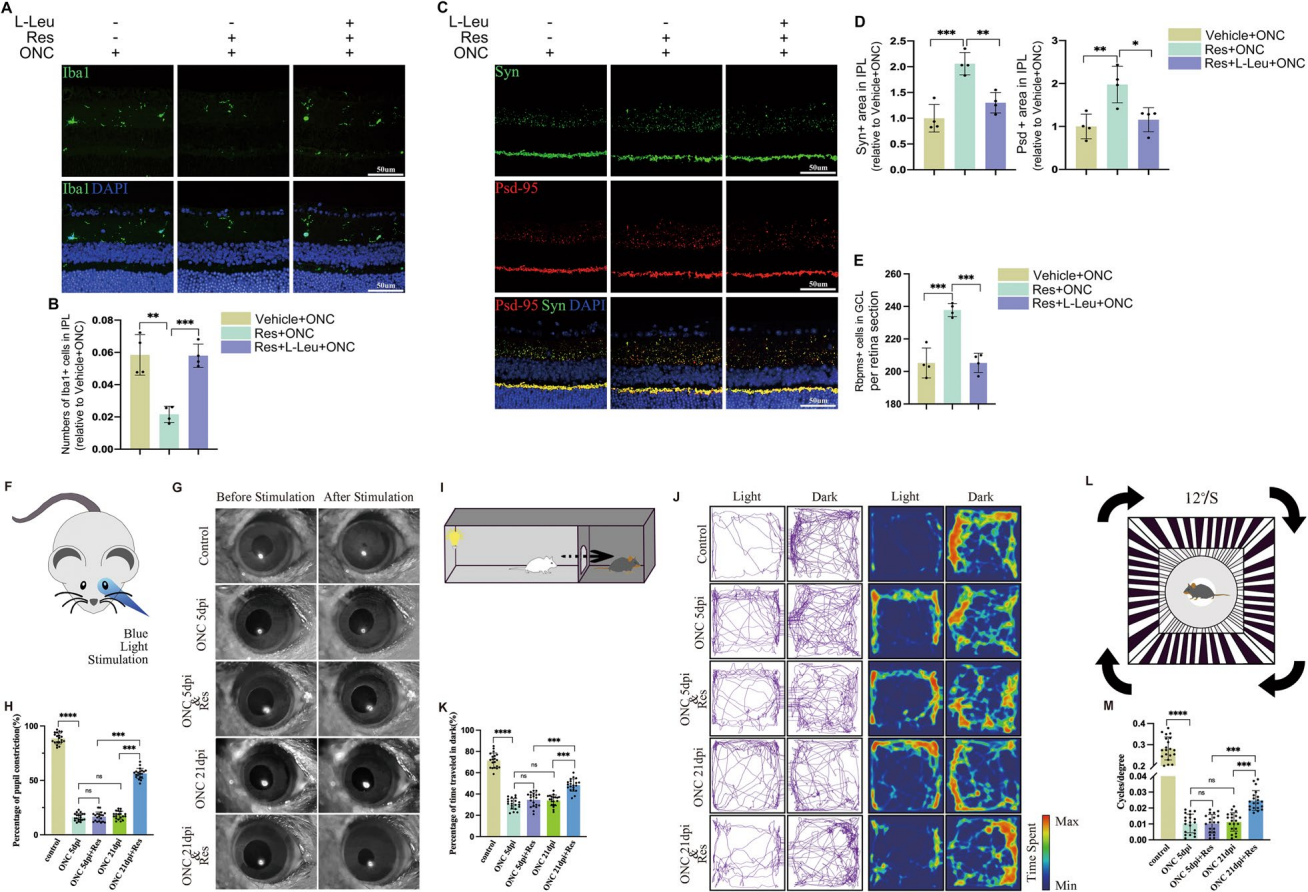
Sirt1-mTORC1 signal pathway is engaged in microglial activation and phagocytose of retinal synapse. **A** mTORC1 activation, induced by l-Leu, reversed the Sirt1-meidated inhibition effect on microglia. Shown are the images of microglial distribution in retinas of ONC mice treated with or without Res or l-Leu. **B** l-Leu mediated mTORC1 activation reversed Sirt1-mediated synapse protection effect. Representative images of retinal synapse of ONC mice with or without Res or L-Leu treatment are presented. **C** Measurement of the number of Iba1 positive cells in Retinal IPL in **A**. **P < 0.01, ***P < 0.001 versus mice treated with Res + ONC. *N* = 4. **D** Quantified measurement of Syn (**E**) and Psd95 (**F**) positive area in retinal IPL in **B**. *P < 0.05, **P < 0.01, ***P < 0.001 versus mice treated with Res + ONC. *N* = 4. **E** Quantification of Rbpms positive cell number in retinal GCL. ***P < 0.001 versus mice treated with Res + ONC. *N* = 4. **F** Schematic diagram illustrating the pupillary light reflex (PLR) test. **G** Representative image of pupil before and after blue light stimulation of different groups. **H** Statistical analysis of mouse pupillary light reflex test. *N* = 20, ****P < 0.0001, **P < 0.01. **I** Schematic diagram illustrating the mouse dark/light preference test. **J** The representative traveling trajectories (left panel) and heat map recordings for time spent in distinct regions of the light–dark box (right panel). **K** Statistical analysis of dark/light preference test. *N* = 20, ****P < 0.0001, **P < 0.01. **L** Schematic diagram illustrating the mouse optomotor response test. **M** Statistical analysis of optomotor response test. *N* = 20, ****P < 0.0001, **P < 0.01

To investigate whether SIRT1 can modulate mTORC1 signal pathway, we further conducted experiments in BV2 cells and mice. Leucine (Leu), implicated in mTORC1 activation, was used as a specific mTORC1 activator [43]. As shown in Additional file 4: Fig. S4A, B, l-Leu could significantly increase the level of phosphorylated S6K, a mTORC1 kinase substrate which could reflect mTORC1 pathway activity. In vitro*,* treating BV2 cells with LPS + IFN-γ resulted in increased level of phosphorylated S6K. However, BV2 cells treated with resveratrol had decreased LPS-induced mTORC1 activation, and Leu overrode the inhibition effect caused by resveratrol (Additional file 4: Fig. S4C, D). Besides, as shown in Additional file 4: Fig. S4C, resveratrol treatment prevented the inflammation factor expression of microglia induced by LPS + IFN-γ. However, when Leu simulation was given, the percentage of expression level increased once more (Additional file 4: Fig. S4E). In vivo, Leu treatment blunted the inhibition effect of resveratrol on microglial activation in vivo (Fig. 6A, C). And the synapse loss in IPL became more severe by Leu treatment post-ONC (Fig. 6B, D). What’s more, Leu also blocked the RGCs body protection effect of resveratrol in mice’s retinas after ONC (Fig. 6E).

Then, we tested the effect of SIRT1 activation on visual function recovery post-ONC. As shown in Fig. 6F, mice were kept in dark condition for one hour and then blue light at 470 nm stimulation was presented to dark-adapted dilated eyes. When compared with Control group, the percentage of pupil constriction decreased significantly in ONC group. SIRT1 activation could led to partial restoration of the pupil reflex at 21-day post injury, although no difference was detected at 5-day post-ONC. And the effect of SIRT1 activation was not related to the time post injury (Fig. 6G, I). Light perception (LP) of mice with bilateral ONC was tested by Dark/light preference tests (Fig. 6I). Decreased duration time in dark chamber was detected in ONC group, which reflected vision loss after injury. And the percentage of time traveled in dark of mice increased at 21-day post-ONC when treated with Res, which indicated that SIRT1 activation could partially promote LP recovery (Fig. 6K). And the preferred movements in dark were more intuitively presented in traveling trajectories and the heat maps (Fig. 6J). Furthermore, to reflect the visual acuity of mice, high contrast visual stimulation was applied to test the optomotor response (Fig. 6L). Higher spatial frequency threshold was detected in mice treated with Res at 21-day post-ONC, which indicated improved visual acuity in this group (Fig. 6M). Overall, visual function was impaired by ONC and could not improve when no treatment applied. SIRT1 activation could attenuate microglial activation and protect neuron at early period post injury, but the function recovery of RGC occurred later.

**Fig. 7:**
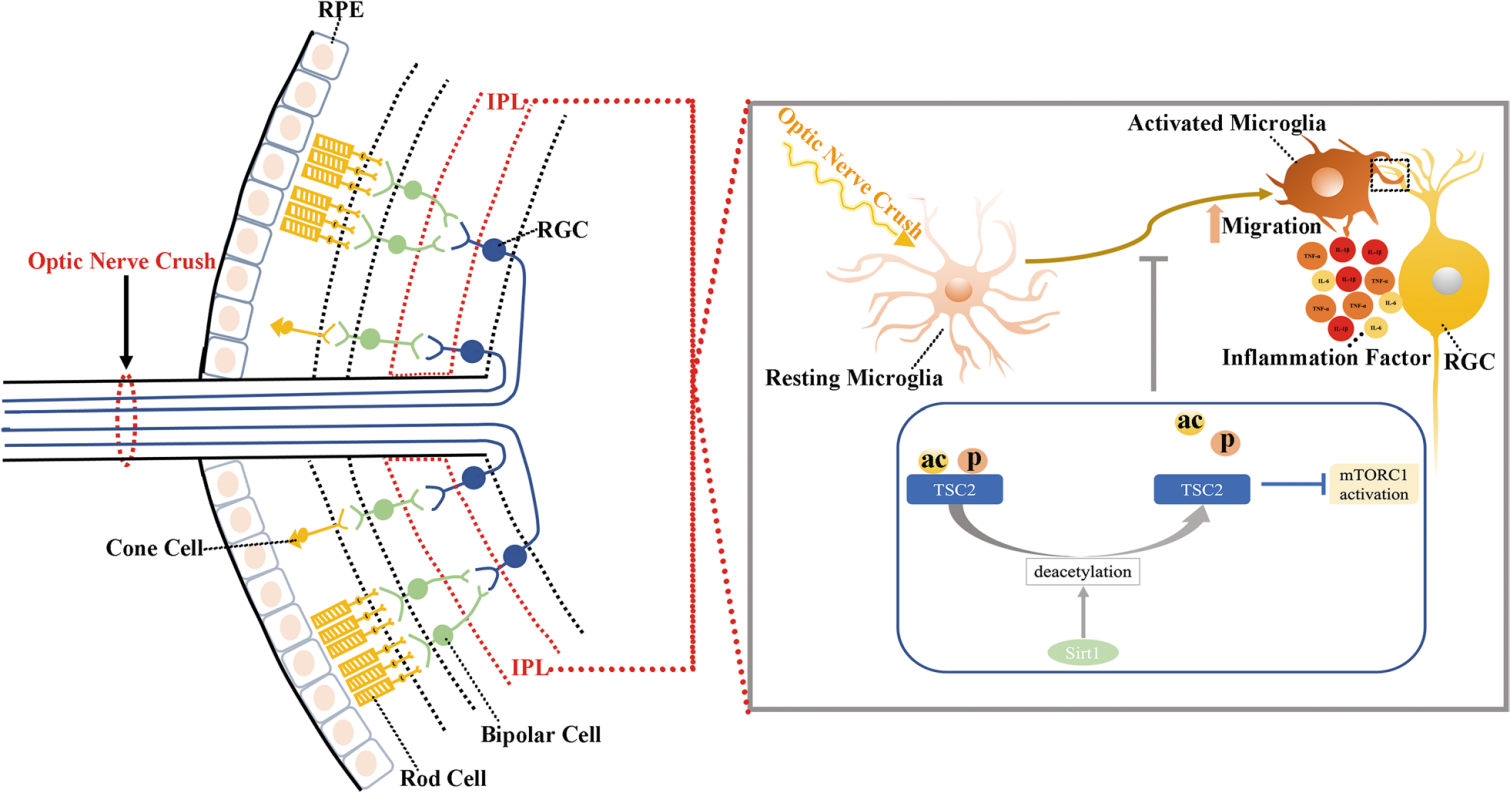
A schematic diagram of proposed crosstalk among Sirt1, TSC2 and mTORC1 in regulation of microglial activation. Microglial activation mediated synapse loss occurs in Retinal IPL after optic nerve injury. In the regulation process of microglial activation, Sirt1 enhances TSC2 activity via deacetylating it at lysine residues 1473 and further reducing its phosphorylation level at tyrosine 1462. Activated TSC2 exerts the inhibition effect on mTORC1. Hence, through the above signaling pathway, microglial Sirt1 could attenuate RGCs injury via inhibiting microglial activation

Taken tother, these data indicate that SIRT1–TSC2–mTORC1 signaling pathway is critical for regulating microglial activation and their phagocytose of RGCs dendrites when ONC is triggered (Fig. 7).

## Discussion

This study demonstrated that ONC in mice causes RGC excitatory synapse damage before soma loss, accompanied by microglia overactivation. SIRT1 was proved to inhibit microglial activation to protect RGCs synapse from being engulfed through mTORC1 signal pathway. We also demonstrated a novel mechanism by which SIRT1 in microglial could mediate deacetylation of TSC2 at lysine residues 1473 to reduce its phosphorylation at tyrosine 1462, which in turn would influence the activity of mTORC1 pathway’s activity.

Microglia are immunocompetent cells that act as neuropathological sensors, and a first line of defense to injuries in the central nervous system (CNS), including the brain and retina [44, 45]. In physiological conditions, microglia survey the retina to detect any damage and respond by combining defensive reactions with neuroprotective activities [48, 49]. When injury or disease stimulus happens, astrocytes, Muller cells, and microglia become activated and release cytokines and chemokines together with subsequent blood-borne macrophage [49]. Among these, microglial activation is detected in early stages of experimental glaucoma retinopathy. It was reported that neurotoxic reactive astrocytes are induced by activated microglia, and this kind of astrocytes would lose phagocytic capacity by in vitro and in vivo experiments [50]. When activated, microglia acquire an amoeboid morphology, accompanied by production of proinflammatory and cytotoxic molecules, such as IL-6, TNF-a and NO [48, 51]. Depletion of microglia with PLX3397, a colony stimulating factor 1 receptor (CSF1R) inhibitor, recovers the cognitive performance in juvenile animals of the Dp (16) mouse model of Down Syndrome [52]. In our previous study, microglial ablation with PLX5622 attenuated RGCs loss after ONC [26]. These findings all pointed to the vital role of microglia in neurodegenerative conditions, especially in early stage. In our research, microglia became activated markedly at 3 days post-ONC in mice, while was still no significant RGCs body loss happened. Activated microglia in INL displayed an amoeboid morphology via retina stretched preparation, and synapses were engulfed by the microglia prior to RGC soma loss. Although previous reports revealed that microglia mediate bone-born monocyte infiltration, the main immune cell react to injury is microglia in early phage post-CNS injury [53]. Hence, Iba1 and Cx3Cre^ER^ were used mainly for detecting and gene manipulation of microglia in our research. These findings suggested microglia was crucial in synapse loss, which integrates neuronal input from photoreceptors to RGC and CNS. Targeting microglia to attenuate synapse loss provided new insights into novel mechanism to prevent vision loss.

Resveratrol and SIRT1 were reported to participate in pathophysiology of degenerative diseases in CNS and retina [50]. During normal aging, SIRT1 is responsible for the maintenance of neural systems and behavior, including the modulation of synaptic plasticity and memory processes [54]. SIRT1 was reported to delay RGC loss following traumatic injury which may include a component of oxidative stress [55]. Besides, the activities of resveratrol against neuroinflammation appear to target activated microglia and result in the reduction of proinflammatory factors (i.e., TNF-α, IL-β, iNOS, etc.). However, the role of resveratrol and SIRT1 in microglia activation and the molecular mechanisms involved are not fully elucidated [56]. Our earlier research discovered important role of microglia SIRT1 in modulating mTORC1 pathway after ONC to attenuate RGC axonal transport. In this study, the SIRT1-mTORC1 pathway showed the same trend in regulating microglia and RGC synapse crosstalk. When we analyzed the mass spectrometry sequencing of acetylated proteins, we found that cells treated with SIRT1-specific inhibitor EX-527 dramatically altered the acetylation TSC2 protein [57]. In in vitro experiments, TSC2 at K1476 was proved to be the vital substrate to influence the activity of mTORC1 pathway through SIRT1 deacetylation modification. These findings all verified SIRT1 to be a promising target for therapy of RGC injury. Previous research reported that SIRT1 has other targets, so SIRT1 may have other downstream to mediate its effect and further research need to be performed [58].

The mTORC1 complex acts as a regulator of cell growth and metabolism through phosphorylation of ribosomal p70S6 kinase and eukaryotic initiation factor 4E binding protein [23]. The inhibition of mTOR can protect neurons from cell death by reducing energy use and mTORC1 was proved to modulate glucose use and glycolysis through Hif1α [59, 60]. Recent research demonstrated that the mTOR pathway in RGCs and other cell types were activated in aged DBA/2J mice eyes [61]. Rapamycin, an mTORC1 inhibitor, can promote cell survival associated with fasting and decreased energy use [60]. Researchers found there are disturbed glucose and pyruvate metabolism in the glaucoma model of DBA/2J mice retina while pyruvate or rapamycin in food showed obvious neuroprotective effect [61]. Also, intraperitoneal injection rapamycin to mice with Sirt1 deletion could partially neutralized inhibitory effects of Sirt1 ablation on longitudinal bone growth, which indicated the causative link between SIRT1 and mTORC1 signaling in the growth plate [25]. Combined with our results, SIRT1, which is mTORC1's upstream regulator, is first linked to regulator of retinal metabolism through control of TSC2 deacetylation.

Protein post-translational modifications (PTMs) introduce structural changes in existing proteins, commonly on the side chains of nucleophilic amino acids, to participate in various biological processes [62, 63]. Common modifications, such as Cys/Lys acylation and Ser/Thr/Tyr phosphorylation, are mediated by enzymes to regulate protein properties, such as enzymatic activity and subcellular localization, etc. [64]. The density of co-occurring protein PTMs can be very high, where multiple PTMs can positively or negatively influence each other (which is termed PTM crosstalk) [65]. For example, activation of p53 was evidenced by its phosphorylation at serine 15 and acetylation at lysine 382 [66]. Acetylation of lysine 382 and phosphorylation of serine392 in p53 could modulate the interaction between p53 and MDC1, a central adaptor that recruits many proteins to sites of DNA damage [67]. In addition to phosphorylation, S6K1 is also regulated by lysine acetylation. Acetylation of the C-terminal region of S6K1 has been reported to inhibit phosphorylation of S6K1 by mTORC1 [24, 68]. Phosphorylation of TSC2 can occur on several residues. It can be phosphorylated and inhibited by Akt and suppresses mTORC1 signaling [69]. Previous research had reported that SIRT1 could regulate TSC2 activation via deacetylation and found that TSC2 K599M variant inhibited mTORC1 activity, while K106Q mutant could lead to mTORC1 signaling activation [70]. However, it remains unclear which amino acid residue of TSC2 that could be deacetylated by SIRT1 still remains unclear. In our study, SIRT1 could deacetylate TSC2 at K1473 while there was no discernible in protein’s expression of TSC2. K1473 mutation would enhance the phosphorylation of T1462 and prevent SIRT1–TSC2 interaction, thus regulating the activity of mTORC1 pathway. This is a novel mechanism to explicate how microglial mTORC1 is regulated by SIRT1, and how phosphorylation and acetylation crosstalk of TSC2 play a significant role in regulating microglial and RGCs synapse remodeling.

## Conclusions

In conclusion, our investigation demonstrates a novel mechanism of the SIRT1–TSC2–mTORC1 pathway in microglial regulation to alert synapse loss, and may provide new insights into ONI disease progression and inform the development of novel therapies to prevent vision loss.

### Supplementary Information

**Additional file 1: Figure S1. **SIRT1 inhibits microglial activation to protect RGCs and synapse from injury. Sirt1 activation could inhibit the expression of proinflammatory factors (TNF-α, iNOS, IL-6) in microglia. The expression level of proinflammatory factors of BV2 cells under Vehicle, LPS + IFN-γ or LPS + IFN-γ + Res treatment. *P < 0.05 versus cells treated with LPS + IFN-γ. *N* = 3. (B and D) LPS and IFN-γ treatment activated BV2 microglia and enhanced its migration ability and phagocytosis, while Res treatment inhibited BV2 cells activation via activating Sirt1. Shown is the images of cells in scratch area of 0 h and 24 h after scratch performed (K), phagocytosis to fluorescent beads of BV2 cells (L). Scale bar, 500 μm for wound-healing assay, 50 μm for beads phagocytosis assay. (C) Measurement of the number of BV2 cells in (B). ***P < 0.001 versus cells treated with LPS + IFN-γ. *N* = 9. (E) Quantification of the number of BV2 cells phagocytosed beads in (D). **P < 0.01 versus cells with LPS + IFN-γ treatment. *N* = 4. (F) Res could effectively enhance SIRT1 activity. Shown is the image of microglial ac-H3K9 level under different treatment. Scale bar, 50 μm. (G) The level of microglial Inos increased after ONC, while SIRT1 activation triggered by Res could inhibit this process, indicating that SIRT1 could inhibit microglial M1 phenotype. Shown is the image of Iba1 and Inos in retina under different treatment. Scale bar, 50 μm.

**Additional file 2: Figure S2. **The neuron protection effect of SIRT1 is mediated by TSC2. (A) Inhibition of TSC2 activity could blunt the inhibition of SIRT1 activation on microglia. Shown are the images of microglial distribution in retinas of ONC mice under different treatment. (B) Quantification of the number of Iba1 + cell in IPL in (A). *N* = 4, *P < 0.01, ***P < 0.001. (C) Inhibition of TSC2 activity could blunt the protection effect of SIRT1 activation on synapse in IPL. Shown are the images of retinal synapse of ONC mice under different treatment. (D) Quantified measurement of Syn (left panel) and Psd95 (right panel) positive area in retinal IPL in C. *N* = 4, ***P < 0.001.

**Additional file 3: Figure S3. **Microglial mTORC1 deficiency inhibits ONC-induced microglial activation. (A) Microglial raptor was knocked out in Raptor^F/F^/Cx3Cr1-Cre^ERT2^ mice with Tamoxifen treatment. Shown is the IB analysis of whole-cell lysates derived from primary brain microglia. (B) Inhibition of mTORC1 activity reduced microglial proinflammatory factors expression. Quantification of the expression level of TNF-α (L), IL-1β (M), IL-6 (*N*). **P < 0.01, ***P < 0.001 versus cells treated with LPS + IFNγ. *N* = 3. (C) Inhibition of mTORC1 activity blunted microglial migration. Images of BV2 cells, treated with or without LPS + IFNγ or Rapa, in the scratch area at 0 h and 24 h after wound-healing assay performed are presented. (D) Quantification of the number of invading BV2 cells in (I). *P < 0.05, ***P < 0.001 versus cells under LPS + IFNγ treatment. *N* = 4. (E) Inhibition of mTORC1 activity attenuated microglial phagocytosis. Shown is the images of CD68 (microglial phagocytosis marker) positive area of BV2 cells with or without LPS + IFNγ or Rapa treatment. (F) Measurement of mean CD68 fluorescent intensity of BV2 cells in (K). **P < 0.01, ***P < 0.001 versus cells under LPS + IFNγ treatment. *N* = 4.

**Additional file 4: Figure S4. **Sirt1-mTORC1 signal pathway is engaged in microglial activation and phagocytose of retinal synapse. (A) The activation of mTORC1 was detected by the phosphorylation level of p70 S6 Kinase, the mTOC1 downstream molecular. L-Leu could activate mTORC1 activity. Shown is the IB analysis of whole-cell lysates derived from BV2 cells with or without L-Leu stimulation. (B) Left panel is the quantification of the p70 S6 Kinase band intensities in (A). ns, no significance. *N* = 4. Right panel is the ratio of the Phospho-p70 S6 Kinase band intensities to p70 S6 Kinase band intensities in (A). ***P < 0.001. *N* = 4. (C) L-Leu could reverse Sirt1 mediated mTOC1 inhibition effect, and both L-Leu and Res treatment had no effect on the expression of p70 S6 Kinase. Shown is the IB analysis of whole-cell lysates derived from BV2 cells under different intervention. (D) Left panel is the quantification of the p70 S6 Kinase band intensities in (G). ns versus Vehicle cells (ANOVA). ns, no significance. *N* = 4. Right panel is the ratio of the Phospho-p70 S6 Kinase band intensities to p70 S6 Kinase band intensities in (G). ***P < 0.001 versus cells treated with LPS + IFNγ, ***P < 0.001 versus cells treated with LPS + IFNγ + Res. *N* = 4. (E) L-Leu could reverse Sirt1 mediated inhibition effect on microglial proinflammatory factors expression. Quantified measurement of the expression level of *TNF-α* (J), *IL-1β* (K), *IL-6* (L). *P < 0.05, ***P < 0.001 versus LPS + IFNγ treating cells, **P < 0.01 versus LPS + IFNγ + Res treating cells. *N* = 4.

## Data Availability

The datasets used and/or analyzed during the current study are available from the corresponding author (Yin Zhao, Email: zhaoyin85@hust.edu.cn) on reasonable request.

## Abbreviations

DEPTOR: DEP domain-containing protein 6
IPL: Inner plexiform layer
Leu: Leucine
mLST8: Mammalian lethal with SEC13 protein 8
mTORC1: Mammalian target of rapamycin complex 1
NAD+: Nicotinamide adenine dinucleotide
ONC: Optic nerve crush
ONI: Optic nerve injury
OPL: Outer plexiform layer
Raptor: Regulatory-associated protein of mTOR
Res: Resveratrol
RGCs: Retinal ganglion cells
Rheb-GTP: GTPase-activating protein for Rheb
S6K1: Ribosomal S6 kinase
SIRT1: Sirtuin 1
TSC1: Tuberous sclerosis complex 1
TSC2: Tuberous sclerosis complex 2
