# Supplementary figures and images for: Microglial SIRT1 activation attenuates synapse loss in retinal inner plexiform layer via mTORC1 inhibition
Source: J Neuroinflammation. 2023 Sep 5;20:202. doi: 10.1186/s12974-023-02886-8 (PMC10481494; doi:10.1186/s12974-023-02886-8)

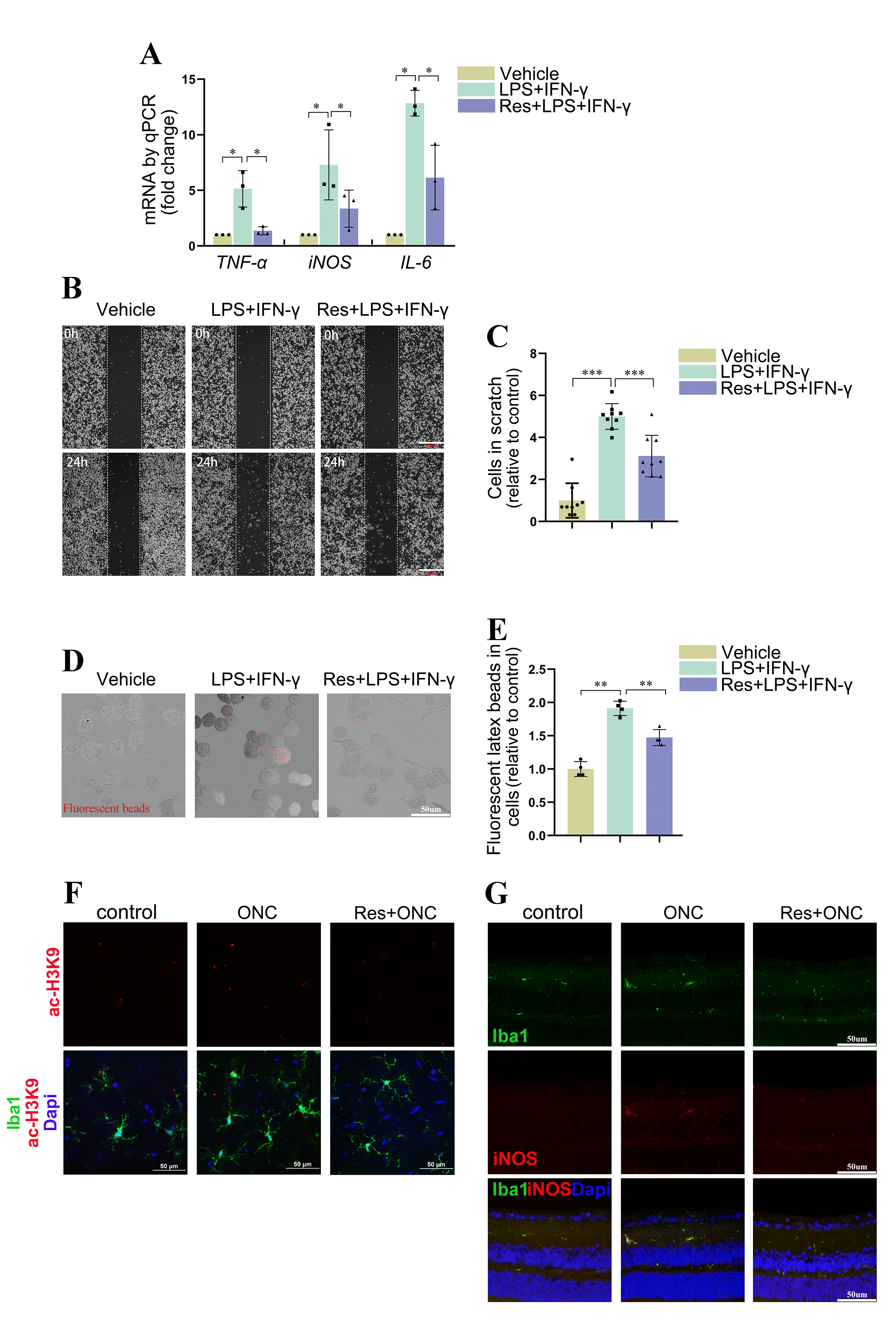

Supplement: Supplementary file 1 — Additional file 1: Figure S1. SIRT1 inhibits microglial activation to protect RGCs and synapse from injury. Sirt1 activation could inhibit the expression of proinflammatory factors (TNF-α, iNOS, IL-6) in microglia. The expression level of proinflammatory factors of BV2 cells under Vehicle, LPS + IFN-γ or LPS + IFN-γ + Res treatment. *P < 0.05 versus cells treated with LPS + IFN-γ. N = 3. (B and D) LPS and IFN-γ treatment activated BV2 microglia and enhanced its migration ability and phagocytosis, while Res treatment inhibited BV2 cells activation via activating Sirt1. Shown is the images of cells in scratch area of 0 h and 24 h after scratch performed (K), phagocytosis to fluorescent beads of BV2 cells (L). Scale bar, 500 μm for wound-healing assay, 50 μm for beads phagocytosis assay. (C) Measurement of the number of BV2 cells in (B). ***P < 0.001 versus cells treated with LPS + IFN-γ. N = 9. (E) Quantification of the number of BV2 cells phagocytosed beads in (D). **P < 0.01 versus cells with LPS + IFN-γ treatment. N = 4. (F) Res could effectively enhance SIRT1 activity. Shown is the image of microglial ac-H3K9 level under different treatment. Scale bar, 50 μm. (G) The level of microglial Inos increased after ONC, while SIRT1 activation triggered by Res could inhibit this process, indicating that SIRT1 could inhibit microglial M1 phenotype. Shown is the image of Iba1 and Inos in retina under different treatment. Scale bar, 50 μm. [file 12974_2023_2886_MOESM1_ESM.tif]

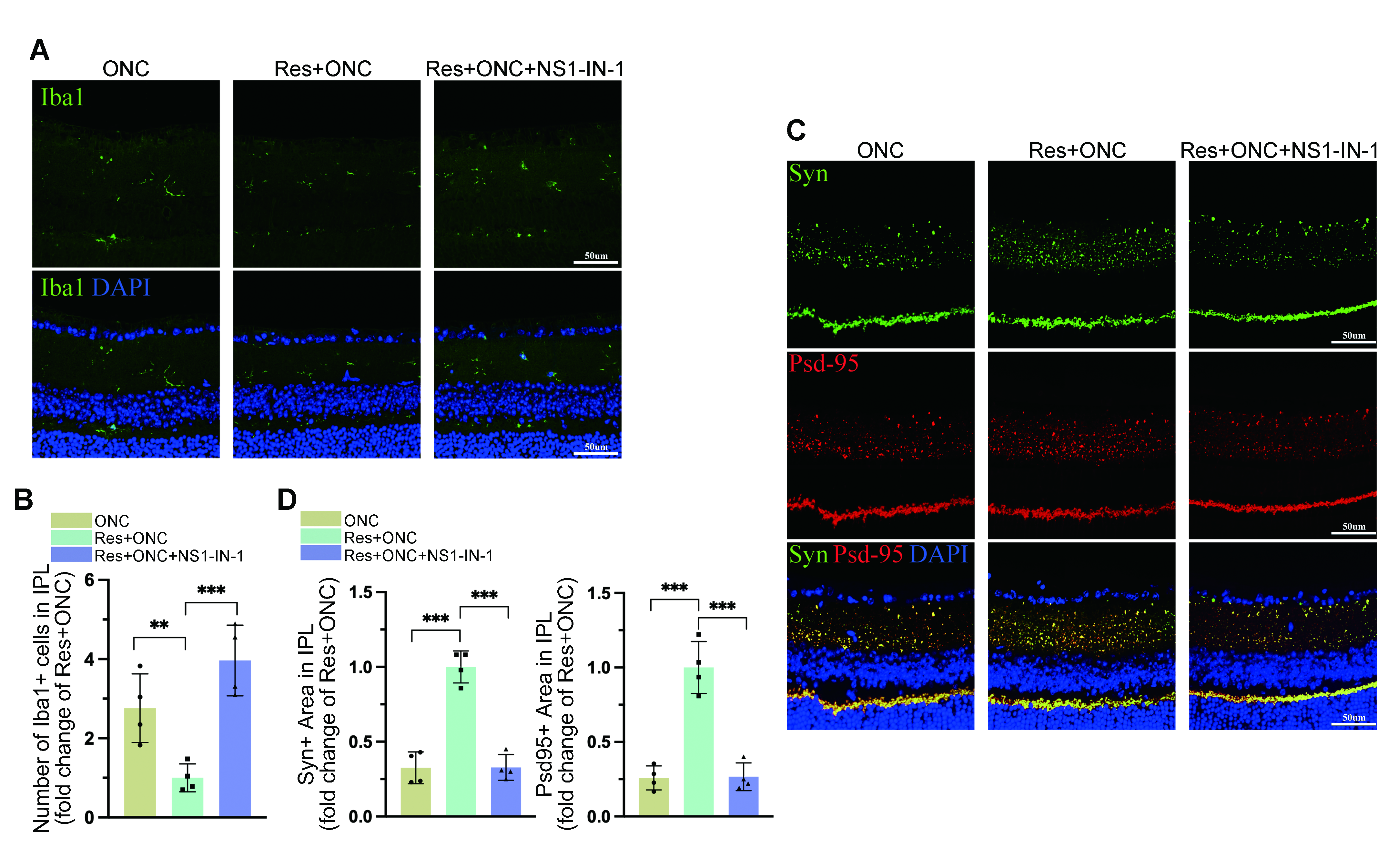

Supplement: Supplementary file 2 — Additional file 2: Figure S2. The neuron protection effect of SIRT1 is mediated by TSC2. (A) Inhibition of TSC2 activity could blunt the inhibition of SIRT1 activation on microglia. Shown are the images of microglial distribution in retinas of ONC mice under different treatment. (B) Quantification of the number of Iba1 + cell in IPL in (A). N = 4, *P < 0.01, ***P < 0.001. (C) Inhibition of TSC2 activity could blunt the protection effect of SIRT1 activation on synapse in IPL. Shown are the images of retinal synapse of ONC mice under different treatment. (D) Quantified measurement of Syn (left panel) and Psd95 (right panel) positive area in retinal IPL in C. N = 4, ***P < 0.001. [file 12974_2023_2886_MOESM2_ESM.tif]

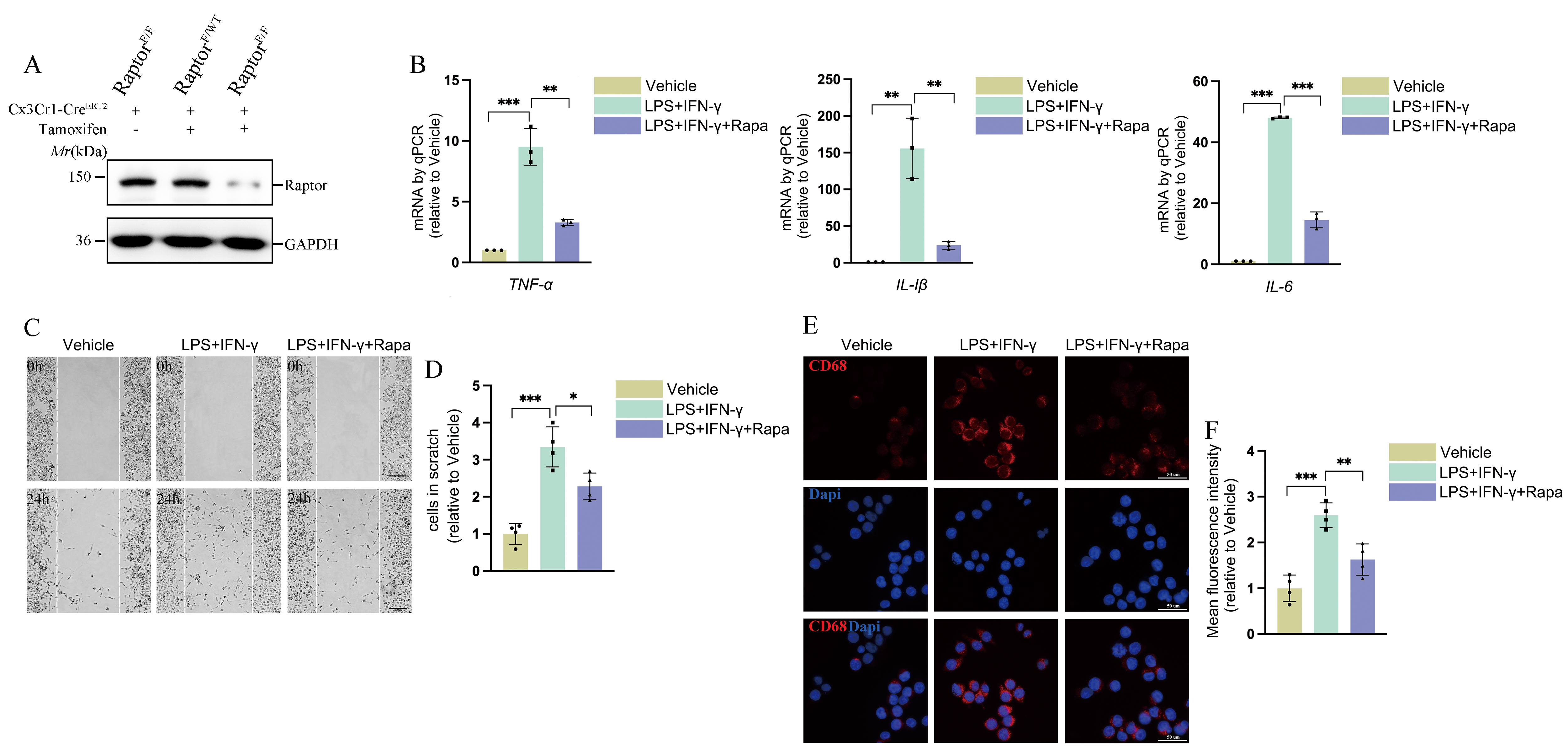

Supplement: Supplementary file 3 — Additional file 3: Figure S3. Microglial mTORC1 deficiency inhibits ONC-induced microglial activation. (A) Microglial raptor was knocked out in RaptorF/F/Cx3Cr1-CreERT2 mice with Tamoxifen treatment. Shown is the IB analysis of whole-cell lysates derived from primary brain microglia. (B) Inhibition of mTORC1 activity reduced microglial proinflammatory factors expression. Quantification of the expression level of TNF-α (L), IL-1β (M), IL-6 (N). **P < 0.01, ***P < 0.001 versus cells treated with LPS + IFNγ. N = 3. (C) Inhibition of mTORC1 activity blunted microglial migration. Images of BV2 cells, treated with or without LPS + IFNγ or Rapa, in the scratch area at 0 h and 24 h after wound-healing assay performed are presented. (D) Quantification of the number of invading BV2 cells in (I). *P < 0.05, ***P < 0.001 versus cells under LPS + IFNγ treatment. N = 4. (E) Inhibition of mTORC1 activity attenuated microglial phagocytosis. Shown is the images of CD68 (microglial phagocytosis marker) positive area of BV2 cells with or without LPS + IFNγ or Rapa treatment. (F) Measurement of mean CD68 fluorescent intensity of BV2 cells in (K). **P < 0.01, ***P < 0.001 versus cells under LPS + IFNγ treatment. N = 4. [file 12974_2023_2886_MOESM3_ESM.tif]

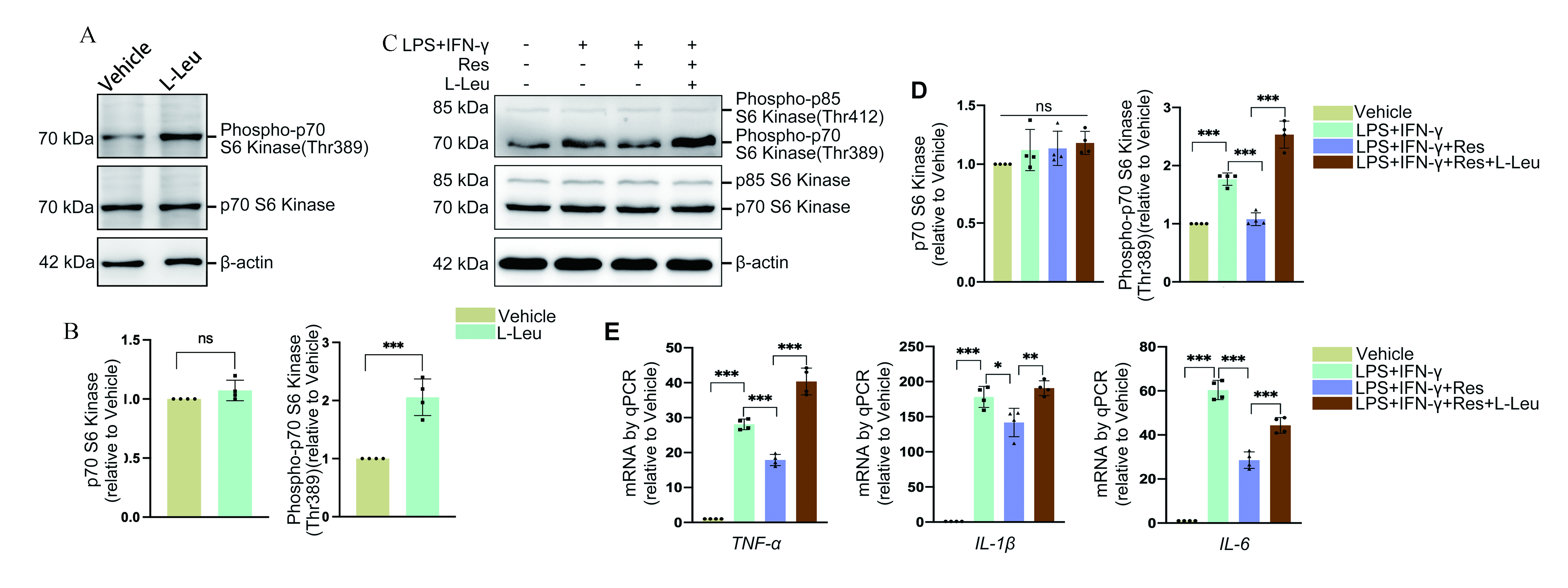

Supplement: Supplementary file 4 — Additional file 4: Figure S4. Sirt1-mTORC1 signal pathway is engaged in microglial activation and phagocytose of retinal synapse. (A) The activation of mTORC1 was detected by the phosphorylation level of p70 S6 Kinase, the mTOC1 downstream molecular. L-Leu could activate mTORC1 activity. Shown is the IB analysis of whole-cell lysates derived from BV2 cells with or without L-Leu stimulation. (B) Left panel is the quantification of the p70 S6 Kinase band intensities in (A). ns, no significance. N = 4. Right panel is the ratio of the Phospho-p70 S6 Kinase band intensities to p70 S6 Kinase band intensities in (A). ***P < 0.001. N = 4. (C) L-Leu could reverse Sirt1 mediated mTOC1 inhibition effect, and both L-Leu and Res treatment had no effect on the expression of p70 S6 Kinase. Shown is the IB analysis of whole-cell lysates derived from BV2 cells under different intervention. (D) Left panel is the quantification of the p70 S6 Kinase band intensities in (G). ns versus Vehicle cells (ANOVA). ns, no significance. N = 4. Right panel is the ratio of the Phospho-p70 S6 Kinase band intensities to p70 S6 Kinase band intensities in (G). ***P < 0.001 versus cells treated with LPS + IFNγ, ***P < 0.001 versus cells treated with LPS + IFNγ + Res. N = 4. (E) L-Leu could reverse Sirt1 mediated inhibition effect on microglial proinflammatory factors expression. Quantified measurement of the expression level of TNF-α (J), IL-1β (K), IL-6 (L). *P < 0.05, ***P < 0.001 versus LPS + IFNγ treating cells, **P < 0.01 versus LPS + IFNγ + Res treating cells. N = 4. [file 12974_2023_2886_MOESM4_ESM.tif]
